# Physical Activity Design Guidelines for School Architecture

**DOI:** 10.1371/journal.pone.0132597

**Published:** 2015-07-31

**Authors:** Jeri Brittin, Dina Sorensen, Matthew Trowbridge, Karen K. Lee, Dieter Breithecker, Leah Frerichs, Terry Huang

**Affiliations:** 1 University of Nebraska Medical Center, College of Public Health, Department of Health Promotion, Social & Behavioral Health, Omaha, NE, United States of America; 2 VMDO Architects, Charlottesville, VA, United States of America; 3 University of Virginia School of Medicine, Department of Emergency Medicine, Charlottesville, VA, United States of America; 4 New York City Department of Health and Mental Hygiene, New York, NY, United States of America; 5 Federal Institute for Posture and Mobilisation Support, Wiesbaden, Germany; 6 University of North Carolina, Chapel Hill, Center for Health Equity, Chapel Hill, NC, United States of America; 7 City University of New York, School of Public Health, New York, NY, United States of America; Arizona State University, UNITED STATES

## Abstract

Increasing children’s physical activity at school is a national focus in the U.S. to address childhood obesity. While research has demonstrated associations between aspects of school environments and students’ physical activity, the literature currently lacks a synthesis of evidence to serve as a practical, spatially-organized resource for school designers and decision-makers, as well as to point to pertinent research opportunities. This paper describes the development of a new practical tool: Physical Activity Design Guidelines for School Architecture. Its aims are to provide architects and designers, as well as school planners, educators, and public health professionals, with strategies for making K-12 school environments conducive to healthy physical activity, and to engage scientists in transdisciplinary perspectives toward improved knowledge of the school environment’s impact. We used a qualitative review process to develop evidence-based and theory-driven school design guidelines that promote increased physical activity among students. The design guidelines include specific strategies in 10 school design domains. Implementation of the guidelines is expected to enable students to adopt healthier physical activity behaviors. The tool bridges a translational gap between research and environmental design practice, and may contribute to setting new industry and education standards.

## Introduction

Physical activity (PA), health, mental alertness, and quality of life are closely interconnected, and the human body needs regular PA in order to function optimally. Evidence is emerging as to the association between children’s PA and academic achievement [1–3], and a substantial body of literature has demonstrated associations between children’s PA and current and future health status, including obesity and related diseases [4]. In the U.S., childhood obesity prevalence tripled between 1980 and 2000 [5], with one-third of U.S. children and youth being overweight or obese today [6]. Concomitantly, few children achieve the current U.S. recommended minimum of 60 minutes per day of moderate to vigorous physical activity (MVPA) [7–9].

In recent years, research on childhood obesity has increasingly focused on transdisciplinary approaches [10], and ecological models with environmental correlates [11], as individually-focused prevention and treatment efforts promoting activity and dietary behavioral change have been difficult to sustain and have had relatively little population-level impact [12,13]. In public health, the built environment has been conceptualized to contain environmental domains—physical, legal, policy, social and cultural—that influence health-related behaviors [14–16]. Theories from several fields of inquiry—including proxemics, architectural theory, environmental psychology, and behavioral geography—have posited that the physical or ‘built’ environment and human behaviors are interrelated, and that physical and social environments are intrinsically linked [17–22]. In addition, social theories have contributed concepts, such as observational learning and environmental determinism, which posit that people can learn new behaviors via exposure to modeling and to environmental change [23,24], and that social structure and human action are interdependent in time and space [25]. Building upon theoretical notions of environment-behavior relationships, studies have focused on the relationships between children’s PA and neighborhood environment characteristics [26], as well as the school classroom environment’s impact on teacher and student behaviors and psychosocial outcomes [27,28]. Past research has indicated that school settings have both direct and mediated impact on learning and achievement outcomes [29,30], and a number of studies have focused on connections between school environmental variables and student learning outcomes [31–36].

Some scientists have suggested that the obesity epidemic is related to “chair-enticing environments,” and have recommended policy changes to promote default PA in school, home and work environments [37]. Interventions to reduce overall time in sedentary behaviors [38], as well as to alter the manner of sedentary time accumulation may be important, as breaks in sedentary behavior have been positively associated with lower body mass index (BMI), and better blood lipids and glucose tolerance [39]. In addition, research has shown that increases in energy expended in everyday activities other than sports-type exercise can impact overall energy balance and can provide protection against fat gain and obesity [40–42]. Environmental design can potentially play a role in supporting such everyday activities.

Based upon associations between aspects of the built environment and health, many have recommended built environment regulatory and non-regulatory policy strategies intended to increase health-promoting behaviors. National and local initiatives are addressing the problem of the U.S. populations’ physical inactivity: “Healthy and safe community environments” is one of four major strategic directions of the National Prevention Strategy, focusing on transforming community settings, including schools, to make healthy choices the “easy” choices. National Prevention Strategy recommendations include integration of health criteria into decision-making across relevant sectors, identifying and implementing proven strategies, and conducting research in areas where evidence is not clear [43]. The City of New York has implemented *Active Design Guidelines* to promote active and healthy living among its residents [44,45]. It has also worked with partners to develop safety strategies for active living [46], and active living housing approaches [47]. The National Collaborative on Childhood Obesity Research (NCCOR), in cooperation with the American Institute of Architects (AIA) and the U.S. Green Building Council (USGBC), has recommended development of evidence-based guidelines for the building industry to promote PA [48]. In partnership with the City of New York, the USGBC has also created a Leadership in Energy and Environmental Design (LEED) green building rating system pilot credit, “Design for Active Occupants,” [49] and is developing an Active Design Index [50].

Schools have been consistently highlighted as important venues for policy-level decisions that impact the health of youth [4,51–54]. A 2012 Institute of Medicine (IOM) report noted that “[c]hildren spend up to half their waking hours in school. In an increasingly sedentary world, schools therefore provide the best opportunity for a population-based approach for increasing PA among the nation’s youth” [55]. Thus, increasing children’s PA in the school environment is now a national priority to address childhood obesity. A 2013 IOM report further emphasized the need to develop high-quality research on the influence of school design on children’s PA and to embrace a “whole-of-school” approach to childhood obesity [4]. Research has indicated that children were sedentary during 70% of class time, including PE class, and that most children also remained sedentary during break and lunchtime [56], highlighting a substantial opportunity to increase PA during the school day. Correlation between school-based physical education (PE) curricula and overall student PA has been documented [57]. Moreover, studies have shown that emphasis of PA in the school curriculum more broadly, i.e., not just in PE class, was beneficial to students’ overall health, social well being, and academic achievement [1,58].

Multi-component, evidence-based school PA interventions, often focusing on PE curricula and including regular activity breaks and family strategies, have been most effective in children [59], but the literature is not clear as to the direct, mediating, or modifying impacts of the built or physical school environment in such interventions. Collaborative work in public health and architecture has pointed to the potential for school design to play a substantial role in obesity prevention [15,60]. However, while there is a growing body of research pertaining to PA-related outcomes and the school physical environment, findings from this work have not been consolidated with the intent of informing school design practice and research.

The billions spent annually in the U.S on public school construction, including new schools, additions, and renovations [61], represent opportunities both to implement evidence-supported health-promoting school designs to reach diverse populations of children, and to develop research opportunities that improve the evidence base. In order to leverage these opportunities, designers and decision-makers need succinct and reliable resources from which to draw, and scientists need to engage in influencing and evaluating the facility-related decisions designers, school administrators, and school communities make.

The *Healthy Eating Design Guidelines for School Architecture* introduced design strategies in school spatial domains to encourage healthy eating behaviors among school communities [62,63]. Here we present a complementary practical synthesis of theory- and evidence-supported school design strategies, in 10 design domains, to promote healthy PA behaviors in school communities. The aims of these *Physical Activity Design Guidelines for School Architecture* are to serve both as a reference for current evidence-supported school design practice to promote PA, and as a source for researchers to generate testable hypotheses for future studies as to the impact of school designs on child and adolescent PA outcomes.

## Methods

### Literature Search

We conducted a comprehensive literature search encompassing K-12 school physical or ‘built’ designs and characteristics, and student PA-related outcomes. Our intention was not to determine or quantify a relationship between a pair of discreetly defined and measured variables, but rather to cover the breadth of research that could have bearing on the development of a translational tool to support both design practitioners and scientists wishing to build upon the evidence base informing PA-promoting school design. We searched the following databases: PubMed/Medline, psycINFO, CINAHL, ERIC, Physical Education Index, Avery Index to Architectural Periodicals, and Educational Administration Abstracts. In PubMed, we employed Medical Subject Headings (MeSH) code, using the following search structure: (Schools[mesh] OR school*) AND (“facility design and construction”[mesh] OR architecture OR “environment design”[mesh] OR “city planning”[mesh] OR “school design” OR “building design” OR “built environment”) AND (exercise[mesh] OR obesity/prevention and control[mesh] OR “health promotion”[mesh] OR “physical activity”). In addition, we conducted a title/abstract [tiab] search of PubMed. For databases not using MeSH, we used a somewhat broader and more simplified keyword structure based on the above, so as to ensure comprehensive coverage of work pertaining to school physical environment variables and PA. Searches included literature through June 2014. One abstract reference was subsequently updated when the full-text article became available [64], and one study in review was subsequently published as an abstract [65]. Additional pertinent references were identified from relevant knowledge domains (e.g., environmental and social psychology, architectural theory, behavioral geography), and in reference lists of individual sources.

**Fig 1:**
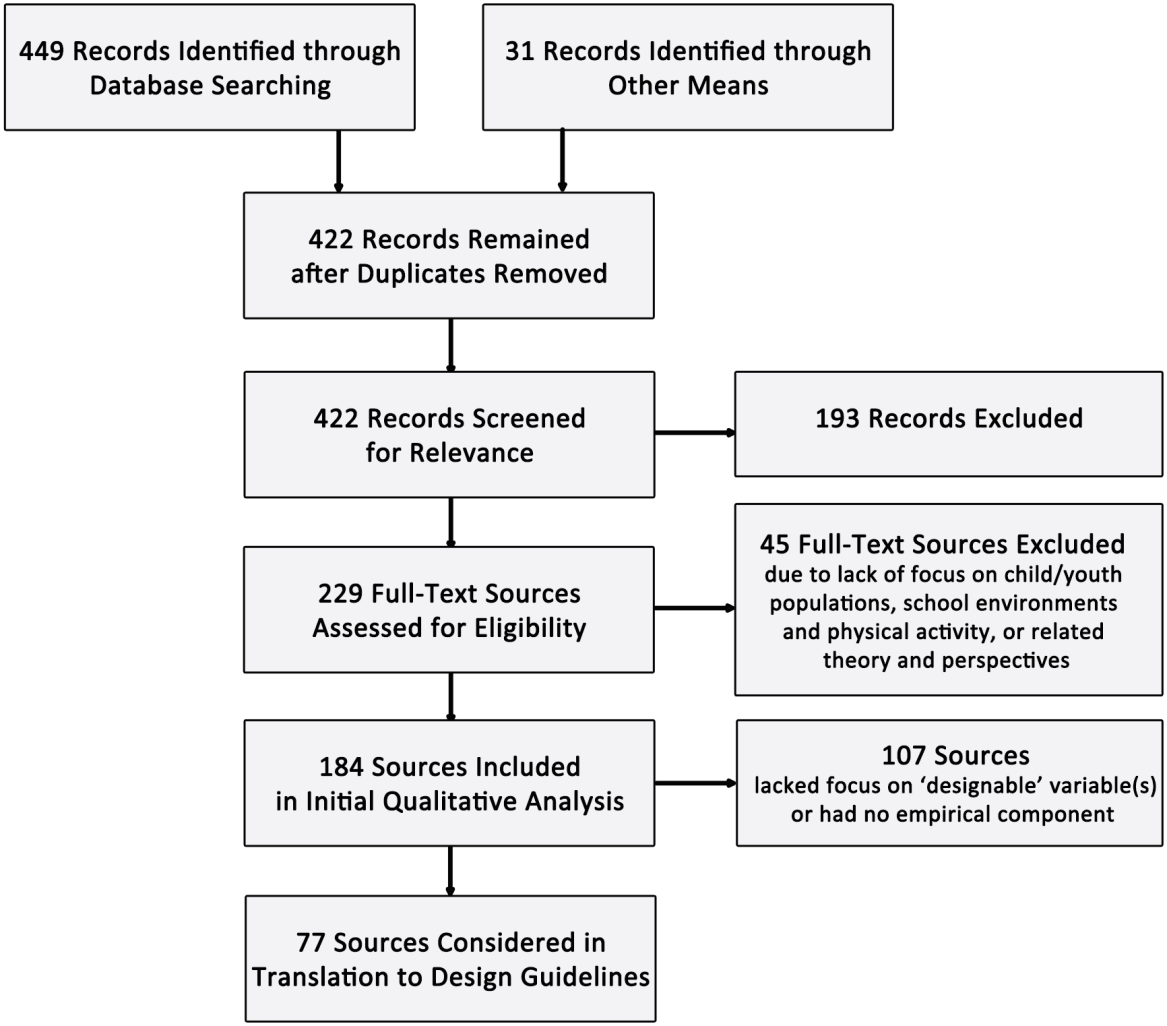
Summary of Source Inclusion/Exclusion Process.

We identified 422 unique sources as potentially relevant to the topic of designing K-12 schools to promote PA. We generally excluded sources that did not pertain to child or adolescent populations, and schools and surrounding environments, unless the work pertained to specific environmental variables or issues of relevance where similar focus on children’s PA and K-12 schools was not available. We included a few studies of preschoolers aged 4 to 6 years, as this age range largely overlaps the age range for Kindergarten and 1^st^ grade in the U.S.; we did not include studies of preschoolers younger than age 4. We also included a few studies in university and other buildings, where environmental variables were of interest, and K-12 school-based studies were not available. In particular, these studies addressed stair usage mainly by adults in several stair intervention scenarios. In order to be inclusive of practice-based outcomes-oriented thinking related to schools, we initially reviewed articles in the architectural literature focusing on learning outcomes in children. However, since these school-related articles did not address PA, they were excluded from the final set of literature. We included one study with the outcome of fat mass index that pertained to active commuting and built environment associations, one study of learning outcomes that were related to school physical environment features and concomitant student PA, and one study of walkability around schools based upon neighborhood-level secondary data. Although we generally limited the search to English-language articles, we included 2 relevant German studies that have not been translated to English. Of 229 full-text sources assessed, we retained 184 for qualitative review. For translation to the design guidelines, we focused on 77 sources that were empirical studies or reviews of empirical work, and that pertained to physical environmental variables that could potentially be designed by practitioners. (Fig 1)

### Transdisciplinary Team and Development of Core Principles

We formed a core team of public health scientists and design practitioners based on the premise that neither group could adequately address development of health-promoting school environments by working solely in disciplinary silos, and with a conviction that there would be benefits to engaging in the challenges of transdisciplinary collaboration. Such challenges have been discussed elsewhere [62]. The review team consisted of professionals in public health academics and practice, and in architectural and interior design, with one team member having formal training in both design and public health research. Team members’ areas of expertise included school architecture and the design of learning environments, the role of PA in healthy childhood development, obesity prevention and intervention research, and designing healthy communities. As a foundation for our intended development of school design guidelines, we formulated a set of core principles as follows:

1. Maximize opportunities for PA (both unintentional and intentional) as part of the school routine.
2. Consider school spaces and features as opportunities to promote children’s natural inclination to move, play, and explore.
3. Apply theory- and evidence-based behavioral science practice to enable the school community to engage in higher levels of default PA.
4. Conceive and articulate school spaces as community assets, and identify nearby community spaces as school assets, to multiply the benefits of school-based healthy PA initiatives.
5. Leverage inherent synergies with current trends in sustainable and universal design, which respectively define good design based on sensitivity to environmental impacts, and accommodation of all user needs and perspectives.

### Review, Synthesis and Translation from Research Findings to the Guidelines

We qualitatively analyzed literature sources to identify source/study types and designs, sample characteristics, approaches and measures, and key findings. Then we engaged in an iterative process of summarizing and synthesizing the findings, assessing relative strengths of evidence, and considering how we might best translate evidence to a structure that would be of practical use both to school designers and to scientists wishing to further knowledge as to health-promoting school environments. We simultaneously asked the questions, “What does the evidence tell us about designing schools to promote PA?” and “What do design practitioners need to know to create schools that promote PA?” We found that the answers to the first question often do not sufficiently answer the second question, supporting a need for both scientists and designers to engage in the other group’s knowledge bases and perspectives. Our ‘translational’ efforts were thus bi-directional, intended not only to translate science to practice, but also to bring practice perspectives to science.

We rated individual studies’ strength of evidence based on research designs and sampling approaches at 3 levels: Strong, Moderate, or Preliminary:

- Strong evidence came from longitudinal cluster randomized or cluster matched controlled trials with measures over time in more than one locale.
- Moderateevidence came from longitudinal approaches with smaller, single-site samples and a comparison or control group, from cross-sectional designs with a large and/or random sample, and reviews consolidating evidence from such studies.
- Preliminary evidence came from single-site longitudinal designs lacking a control or comparison group, and from small pilot cross-sectional associational studies.

Correlates of and causal factors for PA addressed in this set of studies were wide-ranging, sometimes addressed by more than one source, and in a few cases had conflicting results. Therefore, we discussed strength of evidence for the identified environmental variables in terms of their overall support based upon applicable studies.

**Fig 2:**
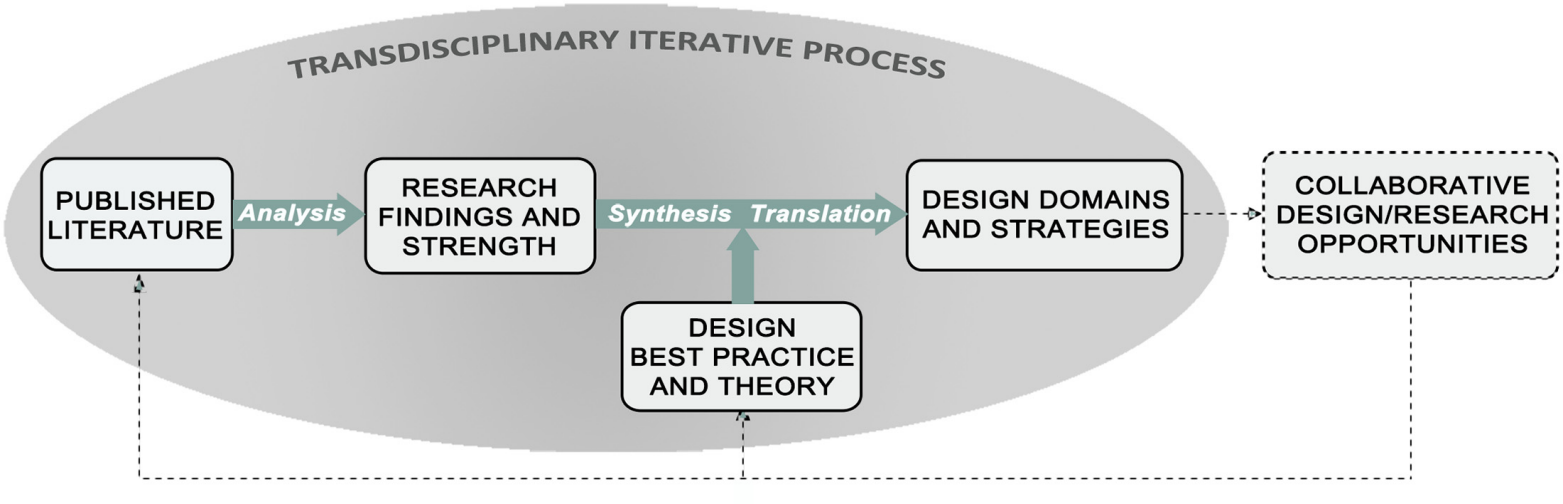
Process Diagram. We reviewed and analyzed literature on the school environment and physical activity to identify research findings and strength of evidence. These findings were then synthesized and translated into a set of design guidelines including spatially-oriented domains and strategies, drawing from best practice and theory where there were gaps in the empirical literature. The guidelines are intended to inform both current practice and collaborative research opportunities that will improve the evidence base.

Once we assessed relative evidentiary strengths, we re-conceptualized these relevant variables into spatially- oriented design domains developed with designers’ input as to their work and decision processes. Typical phases in the building design process have been described elsewhere [62]. Through this work, we considered our core principles, and when empirical research did not definitively or specifically inform needed design knowledge, design best practice and theory-based pathways to impact were also considered as testable hypotheses. (Fig 2)

There were no human subjects in the research presented in this manuscript. The photographs included as illustrations were previously taken by others, are used with their permission, and have been altered to protect all individual identities.

## Results

### Findings from Literature

A 2012 systematic review of literature pertaining to associations between school built environments and the outcome of childhood overweight and obesity (measured as BMI-percentile weight status categories) found very few studies and determined that results were generally inconclusive [66]. There was considerably more literature pertaining to broader PA-related outcomes and the school built environment.

There are many evidence-based PA programs, and such programming in schools has produced increases in children’s time spent in MVPA [59,67], although evidence of impact on weight status remains less clear [68,69]. For the most part, PA program evaluations have not addressed physical school environment variables, but they generally support the need for adequate school PE facilities for in-school and after-school programming, as well as classrooms and other school spaces that can accommodate ample activity and movement among students throughout class time and breaks. In addition, a number of studies have shown that children who walked or cycled to school were more physically active than those who did not actively commute [70–72], and that within-subject time spent in MVPA increased substantially with walking to and from school vs. automobile transport [73]. Children’s independent mobility [74] and active commuting to school have decreased dramatically over past decades [75], and much attention has been paid to active commuting to school as a strategy to increase children’s overall PA levels. Unfortunately, many school and surrounding neighborhood environments have not been conducive to active commuting [76].

Although many of the reviewed studies identified social facilitators and barriers to PA, in addition to physical environment PA correlates, the intentional focus of this review was the physical ‘designed’ environment. It should be noted, though, that in the context of this literature, physical environment impacts on relevant social constructs are both theoretically plausible and likely, and social forces can potentially reinforce or diminish physical environment influences. As examples, teacher presence on playgrounds [77], activity supervision [78], and staff training [79] have been associated with higher MVPA among students, along with various types of fixed and unfixed PA equipment. Here, the specific relationships between equipment and social support were not delineated, but there was indication that teachers reinforced PA opportunities created by elements of the physical environment.

We identified 77 empirical studies and literature reviews that addressed aspect(s) related to school built environment design and students’ PA. This group of literature addressed a broad array of macro- to micro-level school environment characteristics and their relationships to a range of student PA-related measures. For the most part, based upon accepted epidemiological standards, this work has not demonstrated definitive causal associations between school physical environment characteristics and children’s PA. Studies of the impact of environmental settings on human outcomes have presented challenges in control of confounding variables, such as self-selection and spillover effects [80], and it is generally not possible to randomize people to settings such as communities and schools [81]. However, a few studies have used cluster randomized, controlled designs as an achievable alternative to the individual-level randomized controlled trial (RCT).

The final set literature informing the design guidelines consisted of 57 (74.0%) cross-sectional studies, 14 (18.2%) longitudinal designs, and 6 (7.8%) reviews. Of the cross-sectional studies, 54 were quantitative, 1 used mixed methods, and 2 were solely qualitative. One of the qualitative articles was a report of researchers’ observations while conducting a quantitative study rather than a rigorous qualitative design. The mixed methods study and 46 quantitative cross-sectional studies explored potential built environmental correlates of PA. Of the cross-sectional studies, 5 explored the impact of physical environment interventions by comparing different samples at 2 or more points in time. Cross-sectional study sample sizes ranged from 47 to 22,117 individuals. Of the intervention studies with longitudinal measures, 4 were cluster randomized controlled trials, 4 were cluster matched controlled trials, 1 was an individually matched trial, and 5 consisted of within-subject comparisons without randomization or a longitudinal control group. Longitudinal study sample sizes ranged from 9 to 1,465 individuals.

Both independent variable and explanatory built environmental variable definitions and measures varied widely across these studies, precluding opportunities for meta-analyses. PA measures were objectively measured with an instrument or a validated direct observation method in 33 studies, and self- or parent-reported in 24 studies. Among the 25 studies with instrument measures, devices included several types of accelerometers, energy expenditure-measuring armbands, heart rate telemeters, GPS, infrared imagery, and pedometers. Some studies converted raw observed or instrument measures to more clinically-relevant MVPA, and some did not. Even among studies using accelerometers, there were variations in the outcome measures analyzed, including activity counts per time unit, time spent in MVPA or MET-weighted MVPA (MW-MVPA) and other PA intensity levels, and vector magnitude. Other studies measured counts of active users at specified times in defined locations, or assessed proxy reported travel data. See S1 Table for a summary of literature.

The following addresses school built environment PA determinants by relative strength of evidence:

#### Strong to Moderate Evidence

Evidence from 6 studies was deemed strong based upon the defined study design criteria. Of these, 5 focused on school playground interventions, and 1 addressed the student PA impact of school gardens. There was cross-sectional support for the significance of some variables identified in these studies, and also a strong study design of a playground intervention with null results.

Playground Markings and Equipment: A cluster-matched controlled trial at 8 schools in Wales and England found that playgrounds painted with multicolor ground markings—including details such as castles, clock faces, mazes, ladders, letter squares, hopscotch, and animals—increased children’s physical activity levels [82]. An Australian cross-sectional study at 23 primary schools showed that fixed play equipment and painted court and play-line markings were positively associated with MPA, while provision of loose equipment in the playground was associated with more vigorous physical activity (VPA) [77]. A cluster-matched trial at 26 elementary schools in 1 English city showed that playground improvements had significant positive effects on physical activity levels; specifically, play areas were color-coded red for sports, blue for multiple activities, and yellow for quiet play, and included corresponding equipment [83]. A cluster RCT at 7 Belgian elementary schools demonstrated that provision of game equipment during recess increased children’s MVPA [84]. However, another cluster RCT at 40 Belgian public preschools found that introduction of play equipment and playground markings did not impact MVPA [85].

Playground Availability and Safety: Analysis of direct observation data from a cluster-matched controlled trial at 2 New Orleans elementary schools showed that the number of children outdoors and physically active was higher when the school playground was accessible and had supervision, including after school hours. Based on a school-based survey, there was also a decline in students’ sedentary activity with increased playground availability and safety [86]. In a cross-sectional study, focused on adolescent girls, schools with accessible PA facilities outside of school hours were associated with lower BMI but not with time in MVPA [87].

Presence of School Gardens: While a 2007 comprehensive review of research on school gardens found equivocal evidence of school gardens’ impact on student PA [88], a recent cluster RCT in 12 socio-economically and geographically diverse New York State elementary schools showed that installation and use of school gardens induced higher levels of student school-time PA [64].

#### Moderate Evidence

Studies with moderate evidentiary strength denoted other variables related to school grounds.

Presence and Renovation of Schoolyard Playgrounds: The number of permanent playgrounds in schools has been positively associated with MVPA in elementary school students [89]. In a study of twenty urban schoolyards, no particular playground attribute was found to be significantly associated with proportion of active playground users, while the total number of play features and availability of shade were associated with higher utilization [90,91]. Another study evaluating the introduction of renovated schoolyard spaces at Denver schools also found no impact of specific features, although overall utilization increased [92,93].

Outdoor PA Facilities: A study of 130 Norwegian schools showed that students at schools with more outdoor activity facilities reported being significantly more active [94], and another study found that students exhibited the highest levels of PA in an outdoor facility with a handball goal [95]. Positive association between number of active outdoor school facilities and middle school girls’ PA has also been demonstrated [96]. Research on adolescents in 3 U.S. metropolitan areas showed that built-in facilities on the school grounds (e.g., basketball hoops, soccer goal posts, running/walking track) were positively associated with PA [97]. A study of 74 Texas public schools showed that students’ time in MVPA was greater in PE classes held outdoors vs. indoors, generally supporting ample outdoor facilities in school environments [98]. This result corroborated long-established knowledge that children tend to engage in more PA in outdoor vs. indoor environments [99,100]. A UK study also found that the overall number of sports facilities provided at school was positively associated with PA [101], and a U.S. study found association of after-school field accessibility with PA [57]. A California study at 24 schools showed that permanent facilities such as basketball hoops and courts, other sports courts, baseball backstops, etc., along with supervision, were associated with more MVPA [78]. Students’ perceived higher importance of school-based PA facilities and equipment has also been associated with higher PA [102], and provision of PA facilities with recess PA [103].

‘Nature’ in the Schoolyard: A Canadian study, based on a survey of teachers, parents, and school administrators, suggested that school grounds should provide “adequate space, diverse play opportunities, and interaction with natural elements” [104]. A subsequent study by some of the same researchers found that green areas encouraged a high percentage of children toward MPA, vs. a paved, stepped courtyard being associated with high levels of sedentary, seated activity [105]. Another study indicated that schoolyards with ample trees and shrubbery were associated with more PA [106]. Since green school grounds provide opportunities for a greater range of physical activity than the more common asphalt or turf areas, they could play a role in promoting physical activity in children with wide ranging preferences [105]. Supporting this notion was a study comparing PA in 2 independent samples of young children during unstructured recess before and after a schoolyard intervention including a looping cycle path, increased open space in the playground, and a new grass hill. It found fewer sedentary intervals, more intervals in light PA, and higher odds of MVPA in the intervention scenario [107]. The authors recommended environmental changes supporting “novel movement experiences in more expansive spaces” [107].

Schoolyard Surface Materials: Findings regarding surfacing materials were mixed. One study found that both boys’ and girls’ activity levels were higher in soft-surfaced vs. other areas of schoolyards [93], while another study found that MPA was higher on hard-surfaced courts [77]. A study focused on Australian 6^th^ graders showed that grassed surfaces were positively associated with MVPA during recess, but not if shaded [108].

Other studies with moderate evidentiary strength identified PA relationships to school size and PA facilities, and school proximity to other facilities.

School/Campus Size: Larger per student campus and school building areas have been positively associated with PA among students at 10 middle schools [109].

School Indoor PA Facilities: Research on children from disadvantaged backgrounds showed that those attending a school with a gymnasium had more PE time per week than those attending schools without such a facility [110], and a study at 30 Canadian elementary schools showed that students with interschool physical activity programming due to the schools’ lack of adequate facilities engaged in less MVPA [111]. Earlier studies also supported associations between availability of indoor PA facilities at schools and PA outcomes [112]. Some schools have included a gymatorium, in addition to a gymnasium, and instead of a traditional auditorium; a gymatorium has a stage and seating that is flexible or on one side, and provides space for PA when an auditorium is not needed [113]. A combination of recreational equipment and staff training has produced increases in MVPA in elementary school students [79], indicating that activity spaces allowing for active adult supervision may be important.

School Proximity to Other PA Facilities: In a study of adolescent girls, school proximity to recreation facilities was associated with PA [114]. Another study, focused on 12^th^ graders, found that those who attended schools with five or more physical activity facilities within a 0.75 mile buffer zone around the school were more physically active than those attending schools with fewer than 5 nearby physical activity facilities [115].

Many have recommended focus to ensure active commuting to school is safe and convenient [116], and 20 cross-sectional studies addressed active commuting as a means to improve child and adolescent PA. Several inter-related school area environmental constructs emerged from these studies.

Safety: Safety concerns of parents and/or students were major barriers to active commuting [117–122], and Safe Route to School Program sites (created via funding for urban form and safety improvements, such as installation or widening of bicycle lanes, sidewalks, and crosswalks at and near schools) have been associated with higher walking and cycling commuting compared to unimproved sites [123]. In the safety realm, lack of crossing lights [124] and high traffic on the route to school [120,124] also have served as barriers to active commuting. A qualitative study at schools in 7 U.S. states produced similar findings, identifying sidewalks, crosswalks and crossing guards, and sense of personal safety as influential factors in active commuting [125].

Population Density: Some studies noted differences in active commuting behaviors between urban, suburban and rural children, with those in areas of higher population density generally walking more [75,126–128], and those in rural locations more frequently driven to school by parents [129]. Among girls, higher proportion of accessible open land and lower mix of land uses around school were associated with higher fat mass index [130]. Policy recommendations have included moving away from sprawling to more traditional neighborhood plans [131].

Neighborhood Walkability: Several studies showed that neighborhood walkability, a construct encompassing safety, land use, service access, density, and aesthetics, was significantly associated with students’ active commuting [121,132–134]. Research has revealed economic and ethnic disparities in neighborhood walkability [135]. But, while high walkability was associated with more active commuting to school in high-income neighborhoods, it was not related to active commuting in low-income neighborhoods [136]. Those with more active destinations in the neighborhood and more places they enjoyed walking were more likely to commute actively [118].

Distance to School: Studies have shown that distance to school was a barrier to active commuting [117,122], and that those who lived closer to school were more likely to commute actively [118,127,134], in particular if they lived <800 meters from school [124]. In addition, those living closer to school spent more time in MVPA [137]. A Belgian study determined criterion active school commuting distances to be 1.5 kilometers for walking and 3.0 kilometers for bicycling [138].

Connectivity of Route from Home to School: Lack of a direct route to school has been identified as a barrier to active commuting [117]. High route connectivity with low traffic volume was positively associated with walking to school, while regular walking was less likely in areas with high connectivity and high traffic [132]. Retrofitting neighborhoods with walking trails or paths had an impact on neighborhood residents’ PA overall, but was not shown to increase students’ active commuting to school in one study [139].

#### Moderate to Preliminary Evidence

Several studies with moderate and preliminary evidence addressed elements of the school interior and classroom environments.

Open Interior Space and ‘Outside’ Elements: Traditional classrooms with rows of desks and little room or opportunity to move have been the norm for some time in the U.S., but some evidence supports redefining classroom design to support PA and other positive student outcomes. A study of 40 students using within-subject PA measures in a Minnesota city tested the impact of an activity-oriented, open, spacious school environment mimicking the appearance of and called “The Neighborhood.” In this design, representations of environmental elements, such as building facades and a street, were brought to the school interior. The study concluded that children exposed sequentially to 3 distinct school interior environments were more physically active in “The Neighborhood” compared to a traditional school with rows of chairs and desks in the classroom, and compared to a traditional school with stand-biased desks in the classroom [140]. The study also demonstrated cross-sectionally that students in “The Neighborhood” school were just as physically active as other similar students on summer vacation [140].

Flexible ‘Moving’ Classroom: Another study compared students’ PA in ‘moving school’ classrooms at a German school vs. in traditional classrooms at a Belgian school with socio-demographically similar students. The ‘moving’ classrooms were defined by moveable and modular furniture, ample space for frequent and varied in-classroom navigation and movement supported by an activity-promoting school social environment. Findings were that children in the ‘moving’ classrooms were more physically active, and had better posture and lower prevalence of back pain [141].

Stand-biased Desks: A small clustered RCT in 4 classrooms at 1 Texas school found that exposure to stand-biased desks with stools significantly increased class-level energy expenditure [142], and a related study using within-subject measures and no control group found that students’ energy expenditure increased with use of stand-biased desks [143]. A qualitative article about this stand-biased desk intervention reported that students’ focus and attention also improved, and that students generally preferred to stand vs. sit [144]. With adjustments, these desks also supported variations in children’s anthropometry and postures [144], important ergonomic considerations [34,145,146].

Dynamic Furniture: Scientists have argued that the design of a humane working space should consider that bodies, especially growing bodies, are not meant to sit still for long periods of time, and that furniture can support or hinder natural moving behaviors [2,147]. ‘Dynamic furniture’ is designed to foster children’s natural physical movements, and includes pieces such as ergonomic roll-swivel chairs with seat surfaces that move in three dimensions, adjusting to subconscious body position changes and encouraging the body to change positions. Such seating has been shown to have a rhythmic and postural effect, activating the proprioceptive system and improving circulation, raising body temperature [2,148], and improving learning outcomes [2]. A small lab-based study found that children had significantly higher average accelerometer-measured activity counts when using dynamic seating vs. traditional school furniture, although impact on energy expenditure was not detected [65].

Several studies with moderate or preliminary evidentiary strength addressed stair use, mostly among adults. Although stairs tend to be the primary routes of vertical circulation in school environments, some school facilities offer navigation choices between stairs and other routes. Especially among younger student populations, school navigation routes are led by adult teachers, making adult choices potentially relevant.

Stair Spatial Variables: Several spatial variables have been associated with stair use in adults: travel distance from stair to nearest entrance and elevator, occupant load of stair, accessibility of stair, area of visual field from stair, number of turns required for travel from stair to closest entrance, the most integrated path [149], as well as general stair visibility [150].

Stair Prompts: In a study of a clinic, an academic building, and a multi-story housing structure, stair use increased in all settings after posting of stair prompts; at the housing site, stair use remained significantly higher than baseline nine months after the prompts were initially posted [151]. In another study, a motivational component in elevators had no effect on stair use, while the addition of a point-of-choice prompt had a significant effect, indicating that visibility of a prompt at the time of choice encouraged behavior change [152]. In other studies, stair motivational signage was associated with increased stair use [150,153]. A systematic review recommended stair prompts as an evidence-based strategy for increasing stair use [154]. Another review concluded that point-of-choice prompts encouraging stair use can work, although the most effective messages and long-term impact have yet to be determined [155], and others have noted that stronger evidence is desirable [156].

Stair Aesthetics: Use of aesthetic features such as artwork and music were shown to increase use of existing stairs vs. elevators in a limited study in 1 university building [157]. In addition to stair prompts, stair visibility and natural light in stairs have been positively associated with stair use [150].

#### Preliminary Evidence

Work in public health and in human factors engineering has begun to explore use of technologies beyond what is typically available in schools.

Mobile Technologies: Some emerging work has focused on leveraging social marketing in youth PA programs [158], pointing to potential roles for school spaces and mobile and real-time tracking technologies in schools, such as school-based dashboards [159] that could be used to track PA program results in real-time.

Virtual Reality Environments: Recent work has leveraged a virtual reality environment in a school-based PA program. This non-controlled, longitudinal study, called the “American Horsepower Challenge,” produced preliminary evidence that design and integration of a virtual reality environment within the school environment could play a role in increasing youth PA. The program used technology to feed real-world step data from 1,465 middle school students into a virtual designed environment where they could participate in an athletic competition. The virtual environment was intended to motivate all students, even those without particular sports skills, to contribute to winning the competition for their school simply by walking and moving, and participants’ pedometer-measured PA increased significantly over the course of the school program [160].

### Practice-Based Inputs

New York City’s *Active Design Guidelines* were oriented to the perspective of design and spatial decision-making. Some relevant recommended practices applicable to schools and promotion of PA included arranging the building’s program in consideration of the age of users; massing building components in consideration of the scale and age of users and to enhance views of outdoor spaces; providing visually appealing environments along navigation pathways; and allowing for ample daylighting and views to the outdoors from navigation and other areas [45].

Current best practice recommends designing school classrooms to be large enough to accommodate ample movement, to be flexible and mobile in layout to promote activity and accommodate multiple learning and teaching styles, and to make fitness facilities visible (for social modeling) and attractive to reinforce the idea that physical activity is desirable and fun [161]. Architecture and design professionals tend to share and learn best practice via case studies and competitions, and sometimes these are published in architectural and educational journals. This work generally supports school designs that include natural lighting, ample room for movement and flow, and shared community spaces [162]. A subset of the architectural literature on school design is sponsored by industry organizations focused on promoting specific product use in school construction [163,164], highlighting a need for objective and reliable resources for designers.

### Physical Activity Design Guidelines for School Architecture

Children’s school-related PA has been conceptualized previously in categories of commuting PA, recess PA, class PA, and overall PA [165], pointing to potential programmatic intervention areas but not necessarily to built intervention opportunities. To create a tool oriented to the school design process and evaluation of impact on PA outcomes, delineation of domains from a design practice perspective was necessary. Findings from literature suggested that decisions throughout the design process, from school siting, to types and placements of school buildings and PA facilities, to furniture specifications, can be relevant to a health-promoting school. Thus, we organized design strategies into spatially- and process-oriented ‘designable’ domains.

This practical tool, *Physical Activity Design Guidelines for School Architecture*, synthesizes evidence and best practice into strategic actions designers can take in the interest of increasing child and youth PA in and around school settings. The *Guidelines* are intended to be a reference for school designers, educators, and researchers that will evolve with further growth and sophistication of the evidence base. Along with the strategies in each domain, relevant published empirical and review studies are denoted, for those wishing to delve into the nuances of particular studies’ findings, and relative alignments and disagreements. Drawing upon New York City’s definitions and symbols for its *Active Design Guidelines* [65], we rated the substantiality of research-supported evidence for each design strategy as follows:

★ Substantial Evidence—2 longitudinal studies or 5 cross-sectional studies supporting a relationship between the school built environment strategy and PA.
✰ Emerging Evidence—empirical research supporting the strategy exists, but is of a preliminary or pilot nature.
◊ Best Practice—theoretical support and/or practice-based experiential support for the strategy, but no formal evidence base.

The *Guidelines* appear in Table 1.

**Table 1:** Physical Activity Design Guidelines for School Architecture.

Design Domains	Strategies	Relevant Literature	Evidence Rating	Supporting Illustrations
**1 School Siting and Community Connectivity**				
	➢ Consider locating new schools and/or renovating schools in higher density neighborhoods where students live close to school when possible	[75,117,120,126–128,130,134,137]	★	
	➢ Consider safe walking/cycling and public transportation access in choosing school sites	[75,117,118,120–126,132,133,136,138,139]	★	
	➢ Structure built and natural elements on and around the school site for variety and visibility that will be pedestrian-friendly and pedestrian-safe	[105,132]	✰	
	➢ Consider potential cultural, gender, and neighborhood differences in perceptions of safety and aesthetics in potential active commuting routes around schools	[119,120,127,129,135]	✰	
	Connect to existing and/or planned community trail networks, and locate schools near other community and recreational facilities where possible	[114,115,139]	✰	
**2 Building Massing and Programming**				
	➢ Consider age-appropriate scale in massing of building components		◊	
	➢ Consider building connections and spatial patterning as opportunities to promote physical activity		◊	
	➢ Orient building to amplify outdoor views		◊	S1 Fig
	➢ Mass and orient building to allow penetration of natural light from most areas of the building interior		◊	
	➢ Locate building functions to encourage bouts of walking throughout the school day		◊	S2 Fig
	➢ Provide convenient and secure covered bicycle storage on school sites		◊	
	➢ Provide community-use spaces that can accommodate healthy community activities (e.g., local farmer’s market, active participatory events)		◊	S3 and S4 Figs
	Allow for ample school and grounds space per student	[109,128]	✰	
**3 Smart Fitness Facilities**				
	➢ Provide multiple and varied outdoor fitness facilities	[78,87,94,97,101,102,112]	★	
	➢ Include an indoor gymnasium, ideally with an indoor track and ample space to support vigorous PA and PE curricula, especially in locations with frequent inclement weather	[78,87,94,97,101,102,112]	★	
	➢ Provide a ‘gymatorium,’ in addition to a gymnasium, and instead of a traditional auditorium; a gymatorium has a stage and seating that is flexible or on one side, and provides space for PA when an auditorium is not needed		◊	
	➢ Create visibility of fitness and physical activity activities from other parts of the school, such as navigation areas		◊	S5 Fig
	➢ Locate fitness facilities such as gyms and pools centrally if possible for access and visibility		◊	
	➢ Incorporate dedicated interior spaces for a range of types of fitness activities (e.g., smaller, quieter rooms for yoga, Tai chi, etc. in addition to a large gymnasium)		◊	
	➢ Include both soft-surfaced (e.g., soccer/footballs field), and hard-surfaced (e.g., basketball and tennis courts) exterior sports areas	[96,101,112]	✰	
	➢ As sites allow, include hiking and biking trails, and natural areas	[104,107,139]	✰	
	➢ Design indoor and outdoor PA facilities to accommodate use of both fixed and movable equipment	[77,83,89,104,105,107,176]	★	
	➢ Design floor markings that can be used for numerous activities, in addition to using standard court markings in gymnasiums and on hard-surfaced outdoor courts; consider age-appropriateness for types of markings	[77,82]	✰	S6 Fig
	Incorporate natural lighting and outside views from interior facilities and provide visibility to outdoor facilities		◊	
**4 Active Classrooms**				
	➢ Provide ample room for children and teachers to move in and around the classroom, supporting potential activity breaks, as well as PA programs	[140,141]	✰	S7 Fig
	➢ Design modular areas and learning hubs, including activity and reading nooks		◊	
	➢ Provide a flexible classroom layout to allow for multiple and changing configurations	[140,141]	✰	S8 Fig
	➢ Allow space for student-defined learning areas		◊	
	➢ Provide easy access from classrooms to outdoor play and learning areas, especially for young children		◊	
	➢ Provide active time-out space and equipment		◊	
**5 Outdoor Learning Areas**				
	➢ Provide outdoor classroom spaces, with cover and/or shade as appropriate for the local climate	[94,98]	✰	
	➢ Locate outdoor classrooms adjacent to outdoor and natural learning opportunities		◊	S9 Fig
	➢ Include gardens as learning and activity areas, in addition to trails and natural areas	[64,88,105,107]	✰	
	➢ Provide drinking fountains with good-tasting water in outdoor learning areas		◊	
	Provide infrastructure (power, water, lighting) to support high utilization of outdoor classrooms and learning areas		◊	
**6 Active Play and Leisure Areas**				
	➢ Include both hard and soft surfaces, green or ‘natural’ areas, and variations in sun and shade, to promote varieties of activity and exploration of nature in outdoor playground areas	[77,104–107]	★	
	➢ Renovate and/or build playgrounds and break areas to include fixed play equipment with age-appropriate challenge, and less structured space for use of portable equipment	[77,84,89–93,95,103,104]	★	S10 and S11 Figs
	➢ Include multi-color ground markings in playground areas to delineate spaces for many types of activities	[82,83,85]	✰	
	➢ Ensure sufficiently large interior play and gathering areas in regions with frequent inclement weather		◊	
	➢ Provide drinking fountains with good-tasting water in play areas		◊	
	➢ Define arrangements to encourage active adult/supervisor interactions with children in play, recess, and break areas	[83,86,108,176]	✰	
**7 Active Navigation Areas**				
	➢ Locate visually appealing stairs in prominent circulation areas with natural lighting, and place elevators less conspicuously	[149,150,157]	✰	S3 Fig
	➢ Provide alternate routes from place to place where possible		◊	
	➢Provide variation and interest in views (indoor/outdoor) throughout navigation areas and pathways		◊	
	Install features of interest that serve as ‘movement temptations’ in navigation areas to encourage physical interaction with built elements; possibly include elements typically found outdoors	[140]	✰	S12 and S13 Figs
**8 Signage and Wayfinding**				
	➢ Include signage with point of decision prompts for stair use and other PA opportunities	[150–156]	★	
	➢ Develop a wayfinding system that addresses appropriate active navigation (e.g., walking, running) throughout the school and grounds		◊	
	➢ Incorporate educational signage that encourages physical activity, promotes its benefits, and is also age-appropriate and fun		◊	S14 Fig
	➢ Use educational signage to prompt specific physical activity opportunities, beyond stair use		◊	
	Integrate educational signage and wayfinding graphics into the learning curriculum, with potential for social marketing use		◊	S15 Fig
**9 Furniture Specifications**				
	➢ Specify dynamic furniture that is ergonomically appropriate for age, and embraces children’s natural tendency to move and fidget	[2,65,141,148]	✰	S16 Fig
	➢ Specify adjustable, stand-biased desks with stools, and modular furniture, in classrooms	[142–144]	✰	
	➢ Specify a variety of furniture to promote choice options and changes in postures for group work, free work, individual work, etc.		◊	S6 Fig
	➢ Specify furniture with casters to promote agile configurations and novel settings		◊	
**10 Mobile Technologies and Virtual Designed Environments**				
	➢ Incorporate infrastructure for use of technology to promote mobile learning and exploration, and opportunities for health-oriented social marketing fostering PA motivation and competition (e.g., support for school-based mobile devices, real-time feedback dashboards, etc.)		◊	
	Consider designing virtual reality spaces in conjunction with school physical spaces to support PA across the student athletic ability spectrum	[160]	✰	

Evidence Rating Key:

^★^ Substantial Evidence = 2 longitudinal studies or 5 cross-sectional studies supporting a relationship between the school built environment strategy and PA

^✰^ Emerging Evidence = empirical research supporting the strategy exists, but is of a preliminary or pilot nature

^◊^ Best Practice = theoretical support and/or practice-based experiential support for the strategy, but no formal evidence base

(Rating system adopted from the City of New York’s *Active Design Guidelines* [45].)

The 1^st^ domain addresses school siting and connections to community. Its strategies are primarily intended to support students’ active commuting to and from school. The 2^nd^ domain, building massing and programming, has not been addressed in the literature related to PA, but it is an essential and substantial process in designing school environments. Therefore, these strategies largely draw upon best practice, and they are intended to lead designers to consider how massing and programming decisions could impact PA. The 3^rd^ domain addresses school indoor and outdoor fitness facilities, with evidentiary support for specific strategies ranging from substantial empirical evidence to best practice. Empirical studies have pointed to a need for adequate school spaces to integrate physical activity throughout the school day. Although there are few empirical studies of PA directly addressing the 4^th^ domain, classroom design, the strategies presented draw upon this work, as well as encourage spatial designs to accommodate ample movement and activity breaks. Strategies for the 5^th^ domain, outdoor learning areas, draw upon emerging work revealing the benefits of gardens and other outdoor spaces as active learning environments. The 6^th^ domain, active play and leisure areas, draws upon emerging evidence in playground design, and upon theory and best practice. Active navigation areas, the 7^th^ domain, draws upon empirical work along with best practice. The 8^th^ domain, signage and wayfinding, recommends using point-of-decision prompts for stairs and other school-based PA opportunities. In addition, strategies suggest that wayfinding systems developed by designers should encompass PA goals. Specifications for detached furniture are often developed by individuals and/or groups distinct from those who develop the building plan and site, and therefore these strategies are grouped into a 9^th^ domain. Current evidence indicates that dynamic and stand-biased school furnishings could have a positive impact on students’ PA. Finally, the 10^th^ domain, technology and virtual reality environments, builds on emerging work in both public health and human factors engineering. These strategies are intended to prompt school designers to consider potential health impacts of new technologies in the school facility infrastructure, as well as to consider designing virtual environments as extensions of the school educational environment.

### Examples and Supplemental Illustrations

Many of the *Guidelines* have been put into practice at the Carter G. Woodson Education Complex, a primary and elementary school in Buckingham County, Virginia, and at the Fridtjof Nansen School in Hannover, Germany. Visual illustrations of implementations of several design strategies are referenced in Table 1.

## Discussion and Conclusion

The complex causal pathways between environmental factors and human behaviors such as PA are not yet well understood [166,167], but, given need to improve PA behaviors across numerous populations of children, the body of literature associating school environment factors to child and youth PA outcomes is substantial in size and growing. The overall strength of this evidence base remains limited, and longitudinal research of clearly defined variables supporting causal interpretations is warranted. Further explication of built environmental variables and measures, and their causal, mediating, or modifying roles in relation to PA, PA programming, and social environmental variables is needed [96], and ecological models should incorporate context-specific PA and explanatory variable measures [168], as well as strive toward measurement consistency.

One Danish cluster RCT of a multi-component school-based PA intervention—including improvements such as upgrades of outdoor PA areas, construction of leisure areas for adolescents, and improvements in active commuting safety—has reported positive school-time PA effects, but no evidence of impact on students’ overall PA [169,170]. These authors noted that the intervention might have been more successful with more focus on social influences. The study findings raise questions as to the degrees, types, and combinations of built and social environmental factors that could have an appreciable impact. There was little qualitative work in the set of literature reviewed, and rigorous studies including inductive qualitative methods may be useful to inform such understanding of relevant environmental variable definitions and inter-relationships. A mixed-methods longitudinal analysis of the impact of a new school environment on PA outcomes in Buckingham County, Virginia (shown in supplemental illustrations) has yet to be completed, but will be reported when available.

In support of building and evolving school environments to promote PA now, and of growing our knowledge as to the relationships between school environments and PA, the *Physical Activity Design Guidelines for School Architecture* mitigate a sizeable methodological and knowledge gap between PA-focused research and school design practice. As the evidence base around PA and school environments continues to grow, the *Guidelines* will necessarily evolve. As they stand, however, the *Guidelines* contribute substantively to the literature, both as a synthesis of current knowledge and as a practical resource for school designers, decision-makers, and scientists.

The *Guidelines* have several limitations. They draw from a fairly young and undeveloped evidence base, as well as from theory and best practice. Strategies are intended to focus school built environment design decisions on student PA outcomes, but they do not comprise a “formula,” nor do they identify specific design solutions, which eventually must conform to building codes and include numerous details from spatial forms and ordering to material specifications. Potential tensions between strategies, for example, locating schools in denser areas while also providing ample facility space, must necessarily be resolved based upon the context and relative goals of a project. The K-12 population encompasses a wide age range, and all strategies may not necessarily generalize to all ages, geographies, and socio-demographic groups. The strategies focus on elements of the school physical environment and infrastructure that can be designed, but this focus should not preclude explorations of relationships to social environment and infrastructure. It is also not yet clear whether PA behaviors associated with school environment changes may carry over to non-school time, or to other settings later in life. Finally, our literature searches were completed by June 2014, and further work has emerged since this time. However, we have not observed any subsequent studies that would substantially change the content of the *Guidelines*.

In the realm of design practice and school building, the *Guidelines* provide a succinct translation of current evidence to actionable strategies school designers and decision-makers can access and use to orient their work toward desirable PA outcomes. The *Guidelines* can thus function as a component of designers’ ‘toolkit’: The language of the strategies is intended to be specific enough to encourage solutions supporting PA, and at the same time general enough to allow for diverse creative solutions that draw upon local culture and context that may be unique to any given project. The *Guidelines* also provide designers with opportunities to leverage synergies with sustainable practices and universal design. For example, school ground trails, along with a wayfinding and signage system, might incorporate elements of a local ecosystem, and educational point of choice prompts for PA; school garden design could consider how every student, across the spectrum of mobility and ability, would be able to participate in garden activities; and playground design can include multiple structured and unstructured facilities to accommodate and challenge a range of PA abilities. The Guidelines are also flexible enough to help inform school administration and designer decisions, in consideration of evidentiary support, from small-scale renovation to an entirely new site and facility. For example, while school siting may not be relevant to renovation at an existing site, other strategies at a range of scales, from renovating play areas to specifying mobile and dynamic classroom furniture, could well be applied as funding allows. As with any built feature, the costs of construction, maintenance and needed staff support should be considered in light of needs and potential positive health outcomes. Anecdotally, based upon the Virginia school project mentioned above, we have found that focus on health outcomes at the genesis of the school design process resulted in a health-focused facility that cost no more than it would have otherwise.

**Fig 3:**
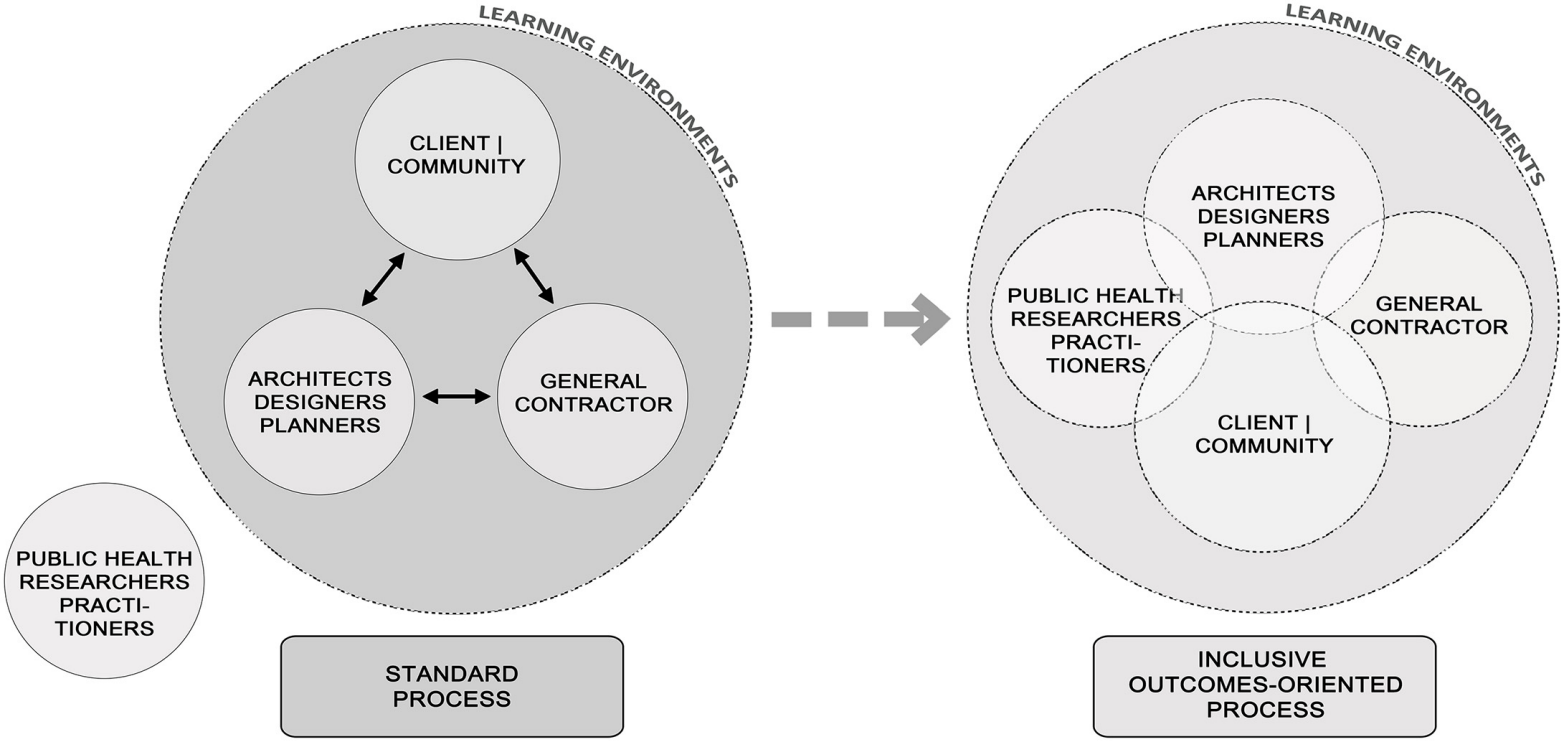
Models of Standard Process and Proposed Transdisciplinary Inclusive Process for Designing and Evaluating Learning Environments.

In the realm of science, the *Guidelines* serve as a structured source for generating testable hypotheses related to school environments and child and youth PA outcomes. Hypotheses could be developed from the *Guidelines* alone, and could also take into account other potential influences. For example, the notion that a built environment change could modify or mediate the effects of a PA program or social intervention could be explored. Such hypotheses can inform future research collaborations, designs and projects that will strengthen the evidence base. It is important to consider research and evaluation opportunities before designing or redesigning a school [156]. The transdisciplinary process we employed was successful in focusing a particular school design project on student’s PA and health outcomes, in conjunction with learning outcomes. We recommend that others consider this transdisciplinary, inclusive model, as illustrated in Fig 3. Public health expertise should be integrated into the learning environments design process from the outset, so that health oriented goals are of primary focus, and so that success in achieving such goals can be rigorously evaluated.

Assessment tools have been developed to reveal issues in community and school environments’ support of PA [171,172], and community-level work has indicated that concerted partnerships focused on designing environments for active living have produced positive results [173–180]. Efforts have emerged to promote health via legislative and funding policies [58,181–188], and researchers have recommended creation of policy on school-community partnerships specifically to promote PA in schools [189]. Others have noted that effective transdisciplinary collaborations are needed [10], including government, corporate, community, and non-profit stakeholders to create health-promoting environments in diverse communities [190]. We expect the *Guidelines* to facilitate focus of industry and education standards on building schools with the goal of improving health outcomes. It is in the interest of the design, school planning, and public health professions, as well as in the interest of communities, to engage in and inform such policy and leadership decisions.

## Supporting Information

S1 Fig Library of the Buckingham County Primary and Elementary Schools at the Carter G. Woodson Education Complex, Dillwyn, Virginia. Much of the school interior includes ample glazing for natural lighting and views of nature. (Photo Credit: Alan Karchmer/VMDO Architects).
(TIF)

S2 Fig First floor and site master plan of the Carter G. Woodson Education Complex, Buckingham County, Virginia. The design promotes bouts of walking during the school day, and includes many varieties of age-appropriate physical activity opportunities. (Image Credit: VMDO Architects/Water Street Studios).
(TIF)

S3 Fig The visually prominent main stairway in the Buckingham County Primary and Elementary Schools at the Carter G. Woodson Education Complex, Dillwyn, Virginia is located near the entry and interior community commons and gathering area. An elevator is available, but located less conspicuously. (Photo Credit: Tom Daly/VMDO Architects).
(TIF)

S4 Fig Community spaces at the in the Buckingham County Primary and Elementary Schools at the Carter G. Woodson Education Complex, Dillwyn, Virginia include a food lab, located in close proximity to the community commons with amphitheatre seating, the dining commons, corner bakery, monumental stair, and entry, all with ample light and outdoor views. (Photo Credit: Alan Karchmer/VMDO Architects).
(TIF)

S5 Fig Drawing upon concepts of observational learning and modeling from social cognitive theory, views from the hallway into the primary school gym in the Buckingham County Primary School at the Carter G. Woodson Education Complex, Dillwyn, Virginia, encourage students to be active. (Photo Credit: Tom Daly/VMDO Architects).
(TIF)

S6 Fig In the gym of the Buckingham County Primary School at the Carter G. Woodson Education Complex, Dillwyn, Virginia, colored floor markings, including wide bands and circles, delineate spaces for various types of simultaneous activities. (Photo Credit: Tom Daly/VMDO Architects).
(TIF)

S7 Fig A classroom at the Fridtjof Nansen School, Hannover, Germany includes mobile, dynamic furniture, allowing flexibility and space to combine active moving with learning. (Photo Credit: Dieter Breithecker/Institute for Posture and Mobilisation Support).
(TIF)

S8 Fig A Kindergarten classroom in the Buckingham County Primary School at the Carter G. Woodson Education Complex, Dillwyn, Virginia includes dynamic seating and trapezoid-shaped tables that adapt to multiple configurations. The classroom also connects directly to an outdoor play area with rain garden features. (Photo Credit: Alan Karchmer/VMDO Architects).
(TIF)

S9 Fig An outdoor classroom at the Buckingham County Primary and Elementary Schools at the Carter G. Woodson Education Complex, Dillwyn, Virginia is adjacent to the vegetable and herb garden, edible orchard, interior dining commons, and kitchen lab. A nature trail that runs throughout the school grounds connects to the garden area. (Rendering Credit: VMDO Architects).
(TIF)

S10 Fig The playground at the Fridtjof Nansen School in Hannover, Germany includes fixed equipment, some of which was built from reclaimed materials, space for moveable equipment and games, and shaded and sunny areas. Water is readily available. Here, the students run up an incline and jump off, enjoying the feeling of weightlessness. (Photo Credit: Dieter Breithecker/Institute for Posture and Mobilisation Support).
(TIF)

S11 Fig The fixed equipment in the playground at the Fridtjof Nansen School in Hannover, Germany is designed for age-appropriate challenge. Here, children organize by way of managing hindrances. (Photo Credit: Dieter Breithecker/Institute for Posture and Mobilisation Support).
(TIF)

S12 Fig The “Tree Canopy” platform designed as a corridor intervention in the Buckingham County Primary School at the Carter G. Woodson Education Complex, Dillwyn, Virginia, is intended to entice interactive and active teaching moments and educates about types of trees native to Virginia. (Photo Credits: Tom Daly (left)/Andrea Hubbell (right)/VMDO Architects).
(TIF)

S13 Fig “Hangelstrecke” play structure encourages bouts of physical activity in a corridor at the Fridtjof Nansen School, Hannover, Germany. (Photo Credit: Dieter Breithecker/Institute for Posture and Mobilisation Support).
(TIF)

S14 Fig Signage throughout the Buckingham County Primary and Elemtary Schools at the Carter G. Woodson Educational Complex, Dillwyn, Virginia educates children about the benefits of being physically active. (Image Credit: VMDO Architects).
(TIF)

S15 Fig The eco-based wayfinding system at the Buckingham County Primary and Elementary Schools at the Carter G. Woodson Educational Complex, Dillwyn, Virginia associates a specific color with each grade level, and engages children to interact visually and physically with educational content. (Image Credit: VMDO Architects).
(TIF)

S16 Fig Open small group learning labs at the Carter G. Woodson Educational Complex in Buckingham County, Virginia include dynamic furniture such as these stools with curved bases. (Photo Credit: Tom Daly/VMDO Architects).
(TIF)

S1 Table Summary of empirical and review literature informing the Physical Activity Design Guidelines for School Architecture, including study design and approach, main findings, and strength of evidence. (DOCX)

## References

[1] Egger J, Bartley K, Benson L, Bellino D, Kerker B. Childhood obesity is a serious concern in new york city: Higher levels of fitness associated with better academic performance. NYC Vital Signs. 2009;8: 1–4.

[2] Dordel S, Breithecker D. Bewegte sSchule als Chance einer Rörderung der Lern- und Leistungsfähigkeit. Haltung und Bewegung. 2003;2: 5–15.

[3] Rasberry CN, Lee SM, Robin L, Laris B, Russell LA, Coyle KK, et al The association between school-based physical activity, including physical education, and academic performance: A systematic review of the literature. Prev Med. 2011;52: S10–S20. 10.1016/j.ypmed.2011.01.027 21291905

[4] Institute of Medicine (US). Committee on Physical Activity and Physical Education in the School Environment Educating the student body: Taking physical activity and physical education to school. Washington, DC: National Academies Press; 2013. 24851299

[5] Benjamin RM. The surgeon general's vision for a healthy and fit nation. Public Health Rep. 2010;125: 514–515. 20597448 10.1177/003335491012500402 PMC2882598

[6] Ogden CL, Carroll MD, Kit BK, Flegal KM. Prevalence of obesity and trends in body mass index among US children and adolescents, 1999–2010. JAMA. 2012;307: 483–490. 10.1001/jama.2012.40 22253364 PMC6362452

[7] Strong WB, Malina RM, Blimkie CJ, Daniels SR, Dishman RK, Gutin B, et al Evidence based physical activity for school-age youth. J Pediatr. 2005;146: 732–737. 15973308 10.1016/j.jpeds.2005.01.055

[8] Physical Activity Guidelines Advisory Committee. Physical activity guidelines advisory committee report, 2008. Washington, DC: US Department of Health and Human Services 2008;2008: A1–H14. 10.1111/j.1753-4887.2008.00136.x 19178654

[9] Andersen LB, Harro M, Sardinha LB, Froberg K, Ekelund U, Brage S, et al Physical activity and clustered cardiovascular risk in children: A cross-sectional study (the European Youth Heart Wtudy). Lancet. 2006;368: 299–304. 16860699 10.1016/S0140-6736(06)69075-2

[10] King AC, Stokols D, Talen E, Brassington GS, Killingsworth R. Theoretical approaches to the promotion of physical activity: Forging a transdisciplinary paradigm. Am J Prev Med. 2002;23: 15–25. 12133734 10.1016/s0749-3797(02)00470-1

[11] Sallis JF, Owen N, Fisher EB. Ecological models of health behavior In: Glanz K, Rimer BK, Viswanath K, editors. Health Behavior and Health Education: Theory, Research, and Practice. San Francisco, CA: John Wiley & Sons; 2008.

[12] Frerichs L, Perin DMP, Huang TTK. Current trends in childhood obesity research. Current Nutrition Reports. 2012;1: 228–238.

[13] Fenton M. Community design and policies for free-range children: Creating environments that support routine physical activity. Childhood Obesity. 2012;8: 44–51. 10.1089/chi.2011.0122 22799480

[14] Buck C, Börnhorst C, Pohlabeln H, Huybrechts I, Pala V, Reisch L, et al Clustering of unhealthy food around German schools and its influence on dietary behavior in school children: A pilot study. Int J Behav Nutr Phys Act. 2013;10. 10.1186/1479-5868-10-65 PMC3686694 23714200

[15] Gorman N, Lackney JA, Rollings K, Huang TT. Designer schools: The role of school space and architecture in obesity prevention. Obes Res. 2007;15: 2521–2530. 10.1038/oby.2007.300 18070739

[16] Huang TT, Drewnosksi A, Kumanyika S, Glass TA. A systems-oriented multilevel framework for addressing obesity in the 21st century. Prev Chronic Dis. 2009;6: A82 19527584 PMC2722412

[17] Hall ET. The Hidden Dimension. New York, NY: Anchor Books; 1969.

[18] Hillier B, Hanson J. The Social Logic of Space. Cambridge: Cambridge University Press; 1984.

[19] Canter D. The facets of place. Advances in Environment, Behavior, and Design. 1997;4: 109–147.

[20] Gump PV. School and classroom environments In: Stokols D, Altman I, editors. Handbook of Environmental Psychology. New York, NY: Wiley; 1987 pp. 691–792.

[21] Ittelson WH. Environment and Cognition. Seminar Press; 1973.

[22] Amedeo DM, Golledge RG. Environmental perception and behavioral geography In: Gaile GL, Willmott CJ, editors. Geography in America at the Dawn of the 21st Century. Oxford: Oxford University Press; 2003 pp. 133–148.

[23] McAlister AL, Perry CL, Parcel GS. How individuals, environments, and health behaviors interact In: Glanz K, Rimer BK, Viswanath K, editors. Health Behavior and Health Education: Theory, Research, and Practice. San Francisco, CA: John Wiley & Sons; 2008.

[24] Bandura A. Social Foundations of Thought and Action: A Social Cognitive Theory. Prentice-Hall, Englewood Cliffs, NJ; 1986.

[25] Giddens A. Central Problems in Social Theory. London: Macmillan; 1979.

[26] Martin JJ, McCaughtry N. Using social cognitive theory to predict physical activity in inner-city African Aamerican school children. J Sport Exerc Psychol. 2008;30: 378–391. 18723898 10.1123/jsep.30.4.378

[27] Weinstein CS. The physical environment of the school: A review of the research. Review of Educational Research. 1979;49: 577–610.

[28] Martin SH. The classroom environment and its effects on the practice of teachers. J Environ Psychol. 2002;22: 139–156.

[29] Moore GT, Lackney JA. Educational facilities for the twenty-first century: Research analysis and design patterns. Center for Architecture and Urban Planning Research, University of Wisconsin-Milwaukee 1994;R94–1.

[30] Martin SH. The classroom environment and its effects on the practice of teachers. J Environ Psychol. 2002;22: 139–156.

[31] Hooper PL, Middleton N, Knuiman M, Giles-Corti B. Measurement error in studies of the built environment: Validating commercial data as objective measures of neighborhood destinations. J Phys Act Health. 2013;10: 792–804. 23074093 10.1123/jpah.10.6.792

[32] Tanner CK. Effects of school design on student outcomes. J Educ Admin. 2009;47: 381–399.

[33] Barrett P, Barrett L. The potential of positive spaces: Senses, brain and spaces. Intelligent Buildings International. 2010;2: 218–228.

[34] Smith TJ. Designing learning environments to promote student learning: Ergonomics in all but name. Work. 2013;44: 39–60. 10.3233/WOR-121493 23241704

[35] Maxwell LE. Home and school density effects on elementary school children the role of spatial density. Environ Behav. 2003;35: 566–578.

[36] Barrett P, Zhang Y, Moffat J, Kobbacy K. A holistic, multi-level analysis identifying the impact of classroom design on pupils' learning. Building and Environment. 2012;59: 678–689.

[37] Levine JA, Vander Weg MW, Hill JO, Klesges RC. Non-exercise activity thermogenesis: The crouching tiger hidden dragon of societal weight gain. Arterioscler Thromb Vasc Biol. 2006;26: 729–736. 16439708 10.1161/01.ATV.0000205848.83210.73

[38] Pate RR, O'Neill JR, Lobelo F. The evolving definition of "sedentary". Exerc Sport Sci Rev. 2008;36: 173–178. 10.1097/JES.0b013e3181877d1a 18815485

[39] Healy GN, Dunstan DW, Salmon J, Cerin E, Shaw JE, Zimmet PZ, et al Breaks in sedentary time: Beneficial associations with metabolic risk. Diabetes Care. 2008;31: 661–666. 10.2337/dc07-2046 18252901

[40] Levine JA. Non-Exercise activity thermogenesis (NEAT). Nutr Rev. 2004;62: S82–S97. 15387473 10.1111/j.1753-4887.2004.tb00094.x

[41] Levine JA, Eberhardt NL, Jensen MD. Role of nonexercise activity thermogenesis in resistance to fat gain in humans. Science. 1999;283: 212–214. 9880251 10.1126/science.283.5399.212

[42] Teske JA, Billington CJ, Kotz CM. Neuropeptidergic mediators of spontaneous physical activity and non-exercise activity thermogenesis. Neuroendocrinology. 2008;87: 71–90. 17984627 10.1159/000110802

[43] National Prevention Council. National prevention strategy healthy and safe community environments. Available: www.surgeongeneral.gov/initiatives/prevention/strategy/healthy-and-safe-community-environments.pdf. 2010; accessed 2014.

[44] Lee KK. Developing and implementing the active design guidelines in New York City. Health Place. 2012;18: 5–7. 10.1016/j.healthplace.2011.09.009 22243901

[45] City of New York. Active design guidelines: Promoting physical activity and health in design; 2010.

[46] Johns Hopkins Center for Injury Research and Policy, NYC Department of Health and Mental Hygiene, Society for Public Health Education. Active design supplement: Promoting safety, version 2. 2013.

[47] Nicoll GA, Lee KK, Dubose J. Active design: Affordable designs for affordable housing. Available: http://herg.gatech.edu/Files/Publications/Affordable-Designs.pdf 2013; accessed 2014.

[48] Trowbridge MJ, Huang TT, Botchwey ND, Fisher TR, Pyke C, Rodgers AB, et al Public health and the green building industry: Partnership opportunities for childhood obesity prevention. Am J Prev Med. 2013;44: 489–495. 10.1016/j.amepre.2013.01.010 23597813

[49] U.S. Green Building Council. LEED design for active occupants. Available: http://www.usgbc.org/node/2648813; accessed 2014.

[50] Lee KK. Developing an active design index for LEED. Green Building Information Gateway. Available: http://insight.gbig.org/developing-an-active-design-index-for-leed/ 2014; accessed 2014.

[51] Ashe M, Graff S, Spector C. Changing places: Policies to make a healthy choice the easy choice. Public Health. 2011;125: 889–895. 10.1016/j.puhe.2011.04.010 21917279

[52] Ashe M, Feldstein LM, Graff S, Kline R, Pinkas D, Zellers L. Local venues for change: Legal strategies for healthy environments. J Law Med Ethics. 2007;35: 138–147. 17341222 10.1111/j.1748-720X.2007.00118.x

[53] Kumanyika SK, Grier S. Targeting interventions for ethnic minority and low-income populations. Future of Children. 2006;16: 187–207. 16532664 10.1353/foc.2006.0005

[54] Hill JO, Wyatt HR, Reed GW, Peters JC. Obesity and the environment: Where do we go from here? Science. 2003;299: 853–855. 12574618 10.1126/science.1079857

[55] Institute of Medicine (US). Committee on Accelerating Progress in Obesity Prevention, Glickman D Accelerating progress in obesity prevention: Solving the weight of the nation. Washington, DC: National Academies Press; 2012, p. 333. 24830053

[56] Nettlefold L, McKay HA, Warburton DE, McGuire KA, Bredin SS, Naylor PJ. The challenge of low physical activity during the school day: At recess, lunch and in physical education. Br J Sports Med. 2011;45: 813–819. 10.1136/bjsm.2009.068072 20215489

[57] Durant N, Harris SK, Doyle S, Person S, Saelens BE, Kerr J, et al Relation of school environment and policy to adolescent physical activity. J Sch Health. 2009;79: 153–159. 10.1111/j.1746-1561.2008.00384.x 19292847

[58] Story M, Nanney MS, Schwartz MB. Schools and obesity prevention: Creating school environments and policies to promote healthy eating and physical activity. Milbank Q. 2009;87: 71–100. 10.1111/j.1468-0009.2009.00548.x 19298416 PMC2879179

[59] Kriemler S, Meyer U, Martin E, van Sluijs EM, Andersen LB, Martin BW. Effect of school-based interventions on physical activity and fitness in children and adolescents: A review of reviews and systematic update. Br J Sports Med. 2011;45: 923–930. 10.1136/bjsports-2011-090186 21836176 PMC3841814

[60] Hanks AS, Just DR, Wansink B. Smarter lunchrooms can address new school lunchroom guidelines and childhood obesity. J Pediatr. 2013;162: 867–869. 10.1016/j.jpeds.2012.12.031 23434267

[61] School Planning and Management. Annual school construction report. Available: http://webspm.com/research/2014/02/annual-school-construction-report/asset.aspx?tc=assetpg&tc=assetpg&returnkey=nwl73OTJUBQwayAH2IzKAv8dRz1cJjD8 2014; accessed 2014.

[62] Huang TT, Sorensen D, Davis S, Frerichs L, Brittin J, Celentano J, et al Healthy eating design guidelines for school architecture. Prev Chron Dis. 2013;10. 10.5888/pcd10.120084 PMC3592783 23449281

[63] Frerichs L, Brittin J, Sorensen D, Trowbridge MJ, Yaroch AL, Siahpush M, et al Influence of school architecture and design on healthy eating: A review of the evidence. Am J Public Health. 2015;105: e46–e57. 10.2105/AJPH.2014.302453 PMC4358206 25713964

[64] Wells NM, Myers BM, Henderson CR. School gardens and physical activity: A randomized controlled trial of low-income elementary schools. Prev Med. 2014;69: S27–S33. 10.1016/j.ypmed.2014.10.012 25456803

[65] Garcia JM, Trowbridge MJ, Huang TT, Weltman A, Sirard JR. Comparison of static and dynamic school furniture on physical activity and learning in children. Med Sci Sport Exerc. 2014;46: 513–514.

[66] Williams AJ, Wyatt KM, Hurst AJ, Williams CA. A systematic review of associations between the primary school built environment and childhood overweight and obesity. Health Place. 2012;18: 504–514. 10.1016/j.healthplace.2012.02.004 22381422

[67] Dobbins M, Husson H, DeCorby K, LaRocca RL. School-based physical activity programs for promoting physical activity and fitness in children and adolescents aged 6 to 18. Cochrane Database Syst Rev. 2013;2. 10.1002/14651858.CD007651.pub2 PMC7197501 23450577

[68] Harris KC, Kuramoto LK, Schulzer M, Retallack JE . Effect of school-based physical activity interventions on body mass index in children: A meta-analysis. CMAJ. 2009;180: 719–726. 10.1503/cmaj.080966 19332753 PMC2659836

[69] Guerra PH, Nobre MR, da Silveira JA. The effect of school-based physical activity interventions on body mass index: A meta-analysis of randomized trials. Clinics. 2013;68: 1263–1273. 10.6061/clinics/2013(09)14 24141844 PMC3782715

[70] Sirard JR, Slater ME. Walking and bicycling to school: A review. Amer J Lifestyle Medicine. 2008;2: 372–396.

[71] Sirard JR, Riner WF Jr, McIver KL, Pate RR. Physical activity and active commuting to elementary school. Med Sci Sports Exerc. 2005;37: 2062–2069. 16331130 10.1249/01.mss.0000179102.17183.6b

[72] Heelan KA, Donnelly JE, Jacobsen DJ, Mayo MS, Washburn R, Greene L. Active commuting to and from school and BMI in elementary school children—preliminary data. Child: Care, Health and Development. 2005;31: 341–349. 10.1111/j.1365-2214.2005.00513.x 15840154

[73] Sirard JR, Alhassan S, Spencer TR, Robinson TN. Changes in physical activity from walking to school. J Nutr Educ Behav. 2008;40: 324–326. 10.1016/j.jneb.2007.12.002 18725153 PMC2556128

[74] Stone MR, Faulkner GE, Mitra R, Buliung RN. The freedom to explore: Examining the influence of independent mobility on weekday, weekend and after-school physical activity behaviour in children living in urban and inner-suburban neighbourhoods of varying socioeconomic status. International J Behav Nutr Phys Act. 2014;11: 5. 10.1186/1479-5868-11-5 PMC3902417 24450739

[75] Buliung RN, Mitra R, Faulkner G. Active school transportation in the greater Toronto area, Canada: An exploration of trends in space and time (1986–2006). Prev Med. 2009;48: 507–512. 10.1016/j.ypmed.2009.03.001 19272403

[76] Kayser B. Determinants of active commuting. Prev Med. 2008;46: 8–8. 17959233 10.1016/j.ypmed.2007.08.016

[77] Willenberg LJ, Ashbolt R, Holland D, Gibbs L, MacDougall C, Garrard J, et al Increasing school playground physical activity: A mixed methods study combining environmental measures and children's perspectives. J Sci Med Sport. 2010;13: 210–216. 10.1016/j.jsams.2009.02.011 19553158

[78] Sallis JF, Conway TL, Prochaska JJ, McKenzie TL, Marshall SJ, Brown M. The association of school environments with youth physical activity. Am J Public Health. 2001;91: 618–620. 11291375 10.2105/ajph.91.4.618 PMC1446652

[79] Huberty JL, Beets MW, Beighle A, Welk G. Environmental modifications to increase physical activity during recess: Preliminary findings from Ready for Recess. J Phys Act Health. 2011;8 Suppl 2: S249–S256. 21918239

[80] Frank LD, Saelens BE, Powell KE, Chapman JE. Stepping towards causation: Do built environments or neighborhood and travel preferences explain physical activity, driving, and obesity? Soc Sci Med. 2007;65: 1898–1914. 17644231 10.1016/j.socscimed.2007.05.053

[81] Nichols A. Evaluation of community-wide interventions. The Urban Institute Available: http://www.urban.org/UploadedPDF/412855-Evaluation-of-Community-Wide-Interventions.pdf 2013; accessed 2014.

[82] Stratton G, Mullan E. The effect of multicolor playground markings on children's physical activity level during recess. Prev Med. 2005;41: 828–833. 16137756 10.1016/j.ypmed.2005.07.009

[83] Ridgers ND, Stratton G, Fairclough SJ, Twisk J. Long-term effects of a playground markings and physical structures on children's recess physical activity levels. Prev Med. 2007;44: 393–397. 17335891 10.1016/j.ypmed.2007.01.009

[84] Verstraete SJ, Cardon GM, De Clercq DL, De Bourdeaudhuij IM. Increasing children's physical activity levels during recess periods in elementary schools: The effects of providing game equipment. Eur J Public Health. 2006;16: 415–419. 16431866 10.1093/eurpub/ckl008

[85] Cardon G, Labarque V, Smits D, De Bourdeaudhuij I. Promoting physical activity at the pre-school playground: The effects of providing markings and play equipment. Prev Med. 2009;48: 335–340. 10.1016/j.ypmed.2009.02.013 19236894

[86] Farley TA, Meriwether RA, Baker ET, Watkins LT, Johnson CC, Webber LS. Safe play spaces to promote physical activity in inner-city children: Results from a pilot study of an environmental intervention. Am J Public Health. 2007;97: 1625–1631. 17666701 10.2105/AJPH.2006.092692 PMC1963283

[87] Scott MM, Cohen DA, Evenson KR, Elder J, Catellier D, Ashwood JS, et al Weekend schoolyard accessibility, physical activity, and obesity: The Trial of Activity in Adolescent Girls (TAAG) study. Prev Med. 2007;44: 398–403. 17292958 10.1016/j.ypmed.2006.12.010 PMC1978099

[88] Ozer EJ. The effects of school gardens on students and schools: Conceptualization and considerations for maximizing healthy development. Health Educ Behav. 2007;34: 846–863. 16861584 10.1177/1090198106289002

[89] Nielsen G, Taylor R, Williams S, Mann J. Permanent play facilities in school playgrounds as a determinant of children's activity. J Phys Act Health. 2010;7: 490–496. 20683091 10.1123/jpah.7.4.490

[90] Colabianchi N, Kinsella AE, Coulton CJ, Moore SM. Utilization and physical activity levels at renovated and unrenovated school playgrounds. Prev Med. 2009;48: 140–143. 10.1016/j.ypmed.2008.11.005 19063915

[91] Colabianchi N, Maslow AL, Swayampakala K. Features and amenities of school playgrounds: A direct observation study of utilization and physical activity levels outside of school time. Int J Behav Nutr Phys Act. 2011;8: 32–32. 10.1186/1479-5868-8-32 21492455 PMC3094267

[92] Anthamatten P, Brink L, Lampe S, Greenwood E, Kingston B, Nigg C. An assessment of schoolyard renovation strategies to encourage children's physical activity. Int J Behav Nutr Phys Act. 2011;8: 27–27. 10.1186/1479-5868-8-27 21477325 PMC3094264

[93] Brink LA, Nigg CR, Lampe SMR, Kingston BA, Mootz AL, van Vliet W. Influence of schoolyard renovations on children's physical activity: The learning landscapes program. Am J Public Health. 2010;100: 1672–1678. 10.2105/AJPH.2009.178939 20634465 PMC2920958

[94] Haug E, Torsheim T, Sallis JF, Samdal O. The characteristics of the outdoor school environment associated with physical activity. Health Educ Res. 2010;25: 248–256. 10.1093/her/cyn050 18936270 PMC2839138

[95] Fjørtoft I, Löfman O, Halvorsen Thorén K. Schoolyard physical activity in 14-year-old adolescents assessed by mobile GPS and heart rate monitoring analysed by GIS. Scand J Public Health. 2010;38: 28–37. 10.1177/1403494810384909 21062837

[96] Cohen D, Scott M, Wang FZ, McKenzie TL, Porter D. School design and physical activity among middle school girls. J Phys Act Health. 2008;5: 719–731. 18820346 10.1123/jpah.5.5.719 PMC3689591

[97] Millstein RA, Strobel J, Kerr J, Sallis JF, Norman GJ, Durant N, et al Home, school, and neighborhood environment factors and youth physical activity. Pediatr Exerc Sci. 2011;23: 487–503. 22109776 10.1123/pes.23.4.487

[98] Skala KA, Springer AE, Sharma SV, Hoelscher DM, Kelder SH. Environmental characteristics and student physical activity in PE class: Findings from two large urban areas of Texas. J Phys Act Health. 2012;9: 481–491. 21934165 10.1123/jpah.9.4.481 PMC3245768

[99] Klesges RC, Eck LH, Hanson CL, Haddock CK, Klesges LM. Effects of obesity, social interactions, and physical environment on physical activity in preschoolers. Health Psychol. 1990;9: 435–449. 2373068 10.1037//0278-6133.9.4.435

[100] Baranowski T, Thompson WO, Durant RH, Baranowski J, Puhl J. Observations on physical activity in physical locations: Ager gender, ethnicity, and month effects. Res Q Exerc Sport. 1993;64: 127–133. 8341835 10.1080/02701367.1993.10608789

[101] van Sluijs E MF, Jones NR, Jones AP, Sharp SJ, Harrison F, Griffin SJ. School-level correlates of physical activity intensity in 10-year-old children. Int J Pediatr Obes. 2011;6: e574–e581. 10.3109/17477166.2010.518239 20854106 PMC3839262

[102] Fein AJ, Plotnikoff RC, Wild TC, Spence JC. Perceived environment and physical activity in youth. Int J Behav Med. 2004;11: 135–142. 15496341 10.1207/s15327558ijbm1103_2

[103] Ridgers ND, Salmon J, Parrish A, Stanley RM, Okely AD. Physical activity during school recess: A systematic review. Am J Prev Med. 2012;43: 320–328. 10.1016/j.amepre.2012.05.019 22898126

[104] Dyment JE, Bell AC. Active by design: Promoting physical activity through school ground greening. Children's Geographies. 2007;5: 463–477.

[105] Dyment JE, Bell AC. Grounds for movement: Green school grounds as sites for promoting physical activity. Health Educ Res. 2008;23: 952–962. 17956885 10.1093/her/cym059

[106] Boldemann C, Blennow M, Dal H, Mårtensson F, Raustorp A, Yuen K, et al Impact of preschool environment upon children's physical activity and sun exposure. Prev Med. 2006;42: 301–308. 16448688 10.1016/j.ypmed.2005.12.006

[107] Nicaise V, Kahan D, Reuben K, Sallis J, F. Evaluation of a redesigned outdoor space on preschool children's physical activity during recess. Pediatr Exerc Sci. 2012;24: 507–518. 23196760 10.1123/pes.24.4.507

[108] Martin K, Bremner A, Salmon J, Rosenberg M, Giles-Corti B. School and individual-level characteristics are associated with children's moderate to vigorous-intensity physical activity during school recess. Aust NZ J Public Health. 2012;36: 469–477. 10.1111/j.1753-6405.2012.00914.x 23025370

[109] Cradock AL, Melly SJ, Allen JG, Morris JS, Gortmaker SL. Characteristics of school campuses and physical activity among youth. Am J Prev Med. 2007;33: 106–113. 17673097 10.1016/j.amepre.2007.04.009 PMC2735893

[110] Fernandes M, Sturm R. Facility provision in elementary schools: Correlates with physical education, recess, and obesity. Prev Med. 2010;50 Suppl 1: S30–S35. 10.1016/j.ypmed.2009.09.022 19850074 PMC2821448

[111] Hobin E, Leatherdale S, Manske S, Dubin J, Elliott S, Veugelers P. A multilevel examination of factors of the school environment and time spent in moderate to vigorous physical activity among a sample of secondary school students in grades 9–12 in Ontario, Canada. Int J Public Health. 2012;57: 699–709. 10.1007/s00038-012-0336-2 22322666

[112] Wechsler H, Devereaux RS, Davis M, Collins J. Using the school environment to promote physical activity and healthy eating. Prev Med. 2000;31: S121–S137.

[113] Doggett F. The evolution of the gymatorium and cafetorium in primary schools. J Acoust Soc Am. 2010;127: 1860–1860.

[114] Scott MM, Evenson KR, Cohen DA, Cox CE. Comparing perceived and objectively measured access to recreational facilities as predictors of physical activity in adolescent girls. J Urban Health. 2007;84: 346–359. 17401691 10.1007/s11524-007-9179-1 PMC2231830

[115] Trilk JL, Ward DS, Dowda M, Pfeiffer KA, Porter DE, Hibbert J, et al Do physical activity facilities near schools affect physical activity in high school girls? Health Place. 2011;17: 651–657. 10.1016/j.healthplace.2011.01.005 21334248 PMC3056935

[116] Wendel AM, Dannenberg AL. Reversing declines in walking and bicycling to school. Prev Med. 2009;48: 513–515. 10.1016/j.ypmed.2009.05.010 19500552

[117] Salmon J, Salmon L, Crawford D, Hume C, Timperio A. Associations among individual, social, and environmental barriers and children's walking or cycling to school. Am J Health Promot. 2007;22: 107–113. 18019887 10.4278/0890-1171-22.2.107

[118] Voorhees CC, Ashwood S, Evenson KR, Sirard JR, Rung AL, Dowda M, et al Neighborhood design and perceptions: Relationship with active commuting. Med Sci Sports Exerc. 2010;42: 1253–1260. 10.1249/MSS.0b013e3181cd5dfd 20019628 PMC2892002

[119] Zhu X, Lee C. Correlates of walking to school and implications for public policies: Survey results from parents of elementary school children in Austin, Texas. J Public Health Policy. 2009;30 Suppl 1: S177–S202. 10.1057/jphp.2008.51 19190573

[120] Silva KS, Vasques DG, Martins CdO, Williams LA, Lopes AS. Active commuting: Prevalence, barriers, and associated variables. J Phys Act Health. 2011;8: 750–757. 21832289 10.1123/jpah.8.6.750

[121] Panter JR, Jones AP, van Sluijs EMF, Griffin SJ. Attitudes, social support and environmental perceptions as predictors of active commuting behaviour in school children. J Epidemiol Community Health. 2010;64: 41–48. 10.1136/jech.2009.086918 19465403 PMC3703574

[122] Heinrich KM, Dierenfield L, Alexander DA, Prose M, Peterson AC. Hawai'i's opportunity for active living advancement (HO'ĀLA): Addressing childhood obesity through safe routes to school. Hawaii Med J. 2011;70: 21–26. 21886289 PMC3158459

[123] Boarnet MG, Anderson CL, Day K, McMillan T, Alfonzo M. Evaluation of the California Safe Routes to School legislation: Urban form changes and children's active transportation to school. Am J Prev Med. 2005;28: 134–140. 15694521 10.1016/j.amepre.2004.10.026

[124] Timperio A, Ball K, Salmon J, Roberts R, Giles-Corti B, Simmons D, et al Personal, family, social, and environmental correlates of active commuting to school. Am J Prev Med. 2006;30: 45–51. 16414423 10.1016/j.amepre.2005.08.047

[125] Eyler AA, Brownson RC, Doescher MP, Evenson KR, Fesperman CE, Litt JS, et al Policies related to active transport to and from school: A multisite case study. Health Educ Res. 2008;23: 963–975. 17956883 10.1093/her/cym061

[126] Mitra R, Buliung RN, Faulkner GEJ. Spatial clustering and the temporal mobility of walking school trips in the greater Toronto area, Canada. Health Place. 2010;16: 646–655. 10.1016/j.healthplace.2010.01.009 20207186

[127] Babey SH, Hastert TA, Huang W, Brown ER. Sociodemographic, family, and environmental factors associated with active commuting to school among US adolescents. J Public Health Policy. 2009;30 Suppl 1: S203–S220. 10.1057/jphp.2008.61 19190574

[128] Braza M, Shoemaker W, Seeley A. Neighborhood design and rates of walking and biking to elementary school in 34 California communities. Am J Health Promot. 2004;19: 128–136. 15559713 10.4278/0890-1171-19.2.128

[129] Loucaides CA. School location and gender differences in personal, social, and environmental correlates of physical activity in Cypriot middle school children. J Phys Act Health. 2009;6: 722–730. 20101915 10.1123/jpah.6.6.722

[130] Harrison F, Jones AP, van Sluijs EM, Cassidy A, Bentham G, Griffin SJ. Environmental correlates of adiposity in 9–10 year old children: Considering home and school neighbourhoods and routes to school. Soc Sci Med. 2011;72: 1411–1419. 10.1016/j.socscimed.2011.02.023 21481505 PMC3759221

[131] Tester JM. The built environment: Designing communities to promote physical activity in children. Pediatrics. 2009;123: 1591–1598. 10.1542/peds.2009-0750 19482771

[132] Giles-Corti B, Wood G, Pikora T, Learnihan V, Bulsara M, Van Niel K, et al School site and the potential to walk to school: The impact of street connectivity and traffic exposure in school neighborhoods. Health Place. 2011;17: 545–550. 10.1016/j.healthplace.2010.12.011 21237697

[133] Panter JR, Jones AP, Van Sluijs E, MF, Griffin SJ. Neighborhood, route, and school environments and children's active commuting. Am J Prev Med. 2010;38: 268–278. 10.1016/j.amepre.2009.10.040 20171528 PMC3819023

[134] Van Dyck D, Cardon G, Deforche B, De Bourdeaudhuij I. Lower neighbourhood walkability and longer distance to school are related to physical activity in Belgian adolescents. Prev Med. 2009;48: 516–518. 10.1016/j.ypmed.2009.03.005 19285102

[135] Zhu X, Lee C. Walkability and safety around elementary schools: Economic and ethnic disparities. Am J Prev Med. 2008;34: 282–290. 10.1016/j.amepre.2008.01.024 18374241

[136] Kerr J, Rosenberg D, Sallis JF, Saelens BE, Frank LD, Conway TL. Active commuting to school: Associations with environment and parental concerns. Med Sci Sports Exerc. 2006;38: 787–794. 16679998 10.1249/01.mss.0000210208.63565.73

[137] Cohen DA, Ashwood JS, Scott MM, Overton A, Evenson KR, Voorhees CC, et al Proximity to school and physical activity among middle school girls. J Phys Act Health. 2006;3: S129–S138. 28834509 10.1123/jpah.3.s1.s129

[138] D'Haese S, De Meester F, De Bourdeaudhuij I, Deforche B, Cardon G. Criterion distances and environmental correlates of active commuting to school in children. Int J Behav Nutr Phys Act. 2011;8: 88–88. 10.1186/1479-5868-8-88 21831276 PMC3168397

[139] Fitzhugh EC, Bassett DR, Evans MF. Urban trails and physical activity: A natural experiment. Am J Prev Med. 2010;39: 259–262. 10.1016/j.amepre.2010.05.010 20709258

[140] Lanningham-Foster L, Foster RC, McCrady SK, Manohar CU, Jensen TB, Mitre NG, et al Changing the school environment to increase physical activity in children. Obesity. 2008;16: 1849–1853. 10.1038/oby.2008.282 18535550 PMC2690697

[141] Cardon G, De Clercq D, De Bourdeaudhuij I, Breithecker D. Sitting habits in elementary schoolchildren: A traditional versus a "moving school". Patient Educ Couns. 2004;54: 133–142. 15288906 10.1016/S0738-3991(03)00215-5

[142] Benden ME, Blake JJ, Wendel ML, Huber JC Jr. The impact of stand-biased desks in classrooms on calorie expenditure in children. Am J Public Health. 2011;101: 1433–1436. 10.2105/AJPH.2010.300072 21421945 PMC3134494

[143] Benden ME, Wendel ML, Jeffrey CE, Zhao H, Morales ML. Within-subjects analysis of the effects of a stand-biased classroom intervention on energy expenditure. J Exerc Physiology. 2012;15: 9–19.

[144] Blake JJ, Benden ME, Wendel ML. Using stand/sit workstations in classrooms: Lessons learned from a pilot study in Texas. J Public Health Manag Pract. 2012;18: 412–415. 10.1097/PHH.0b013e3182215048 22836531

[145] Gouvali MK, Boudolos K. Match between school furniture dimensions and children's anthropometry. Appl Ergon. 2006;37: 765–773. 16442494 10.1016/j.apergo.2005.11.009

[146] Schröder I. Variations of sitting posture and physical activity in different types of school furniture. Coll Antropol. 1997;21: 397–403. 9439057

[147] Breithecker D. Physically active schoolchildren—alert heads Teaching with exercise. Opportunities to improve performance and the ability to study? Wiesbaden, Germany: Federal Working Group on the Development of Posture and Exercise. n.d.

[148] Ludwig O, Breithecker D. Untersuchung zur Änderung der Oberkörperdurchblutung während des Sitzens auf Stühlen mit beweglicher Sitzfläche. Haltung und Bewegung. 2008;3: 5–12.

[149] Nicoll G. Spatial measures associated with stair use. Am J Health Promot. 2007;21: 346–352. 17465180 10.4278/0890-1171-21.4s.346

[150] Ruff RR, Rosenblum R, Fischer S, Meghani H, Adamic J, Lee KK. Associations between building design, point-of-decision stair prompts, and stair use in urban worksites. Prev Med. 2014;60: 60–64. 10.1016/j.ypmed.2013.12.006 24355575

[151] Lee KK, Perry AS, Wolf SA, Agarwal R, Rosenblum R, Fischer S, et al Promoting routine stair use: Evaluating the impact of a stair prompt across buildings. Am J Prev Med. 2012;42: 136–141. 10.1016/j.amepre.2011.10.005 22261209

[152] Lewis A, Eves F. Prompt before the choice is made: Effects of a stair-climbing intervention in university buildings. Br J Health Psychol. 2012;17: 631–643. 10.1111/j.2044-8287.2011.02060.x 22248016

[153] Ford MA, Torok D. Motivational signage increases physical activity on a college campus. J Am Coll Health. 2008;57: 242–244. 10.3200/JACH.57.2.242-244 18809541

[154] Community Preventive Services Task Force. The guide to community preventive services: Environmental and policy approaches to physical activity: Point-of-decision prompts to encourage use of stairs. Available: www.thecommunityguide.org/pa/environmental-policy/podp.html. 2005; accessed 2014.

[155] Nocon M, Muller-Riemenschneider F, Nitzschke K, Willich SN. Review article: Increasing physical activity with point-of-choice prompts: A systematic review. Scand J Public Health. 2010;38: 633–638. 10.1177/1403494810375865 20601438

[156] Zimring C, Joseph A, Nicoll GL, Tsepas S. Influences of building design and site design on physical activity research and intervention opportunities. Am J Prev Med. 2005;28: 186–193. 15694527 10.1016/j.amepre.2004.10.025

[157] Boutelle KN, Jeffery RW, Murray DM, Schmitz KH. Using signs, artwork, and music to promote stair use in a public building. Am J Public Health. 2001;91: 2004–2006. 11726383 10.2105/ajph.91.12.2004 PMC1446922

[158] Thompson D, Cantu D, Bhatt R, Baranowski T, Rodgers W, Jago R, et al Texting to increase physical activity among teenagers (TXT me!): Rationale, design, and methods proposal. JMIR Res Protoc. 2014;3: e14 10.2196/resprot.3074 24622344 PMC3967196

[159] Maldonado RM, Kay J, Yacef K, Schwendimann B. An interactive teacher’s dashboard for monitoring groups in a multi-tabletop learning environment. Intelligent Tutoring Systems. 2012: 482–492.

[160] Poole ES, Miller AD, Xu Y, Eiriksdottir E, Catrambone R, Mynatt ED. The place for ubiquitous computing in schools: Lessons learned from a school-based intervention for youth physical activity. Proceedings of the 13th international conference on Ubiquitous computing. 2011: 395–404.

[161] The Third Teacher: OWP/P Cannon Design, VS Furniture, Bruce Mau Design; 2010.

[162] Hagle M. A school design primer: What are the lessons learned from new schools funded by the 2007 HISD bond? Arch Design Rev Houston. 2013;92: 20–29.

[163] Carpet and Rug Institute. Carpet for schools: A sustainable solution that enhances learning and health. Architectural Record. 2010;198: 123–127.

[164] Professional Glidden. Functional color and design in education environments: Smart choices in color and design facilitate the learning process. Architectural Record. 2013;201: 262–265.

[165] Harrison F, Jones AP. A framework for understanding school based physical environmental influences on childhood obesity. Health Place. 2012;18: 639–648. 10.1016/j.healthplace.2011.12.009 22281440 PMC3759222

[166] Handy SL, Boarnet MG, Ewing R, Killingsworth RE. How the built environment affects physical activity: Views from urban planning. Am J Prev Med. 2002;23: 64–73. 12133739 10.1016/s0749-3797(02)00475-0

[167] Bauman AE, Sallis JF, Dzewaltowski DA, Owen N. Toward a better understanding of the influences on physical activity: The role of determinants, correlates, causal variables, mediators, moderators, and confounders. Am J Prev Med. 2002;23: 5–14. 12133733 10.1016/s0749-3797(02)00469-5

[168] Giles-Corti B, Timperio A, Bull F, Pikora T. Understanding physical activity environmental correlates: Increased specificity for ecological models. Exerc Sport Sci Rev. 2005;33: 175–181. 16239834 10.1097/00003677-200510000-00005

[169] Toftager M, Christiansen LB, Ersbøll AK, Kristensen PL, Due P, Troelsen J. Intervention effects on adolescent physical activity in the multicomponent SPACE study: A cluster randomized controlled trial. PloS ONE. 2014;9: e99369 10.1371/journal.pone.0099369 24921948 PMC4055775

[170] Toftager M, Christiansen LB, Kristensen PL, Troelsen J. SPACE for physical activity—a multicomponent intervention study: Study design and baseline findings from a cluster randomized controlled trial. BMC Public Health. 2011;11: 777–777. 10.1186/1471-2458-11-777 21985278 PMC3202239

[171] Kim S, Adamson KC, Balfanz DR, Brownson RC, Wiecha JL, Shepard D, et al Development of the community healthy living index: A tool to foster healthy environments for the prevention of obesity and chronic disease. Prev Med. 2010;50 Suppl 1: S80–S85. 10.1016/j.ypmed.2009.07.025 19744511

[172] Jones NR, Jones A, van Sluijs E,M.F., Panter J, Harrison F, Griffin SJ. School environments and physical activity: The development and testing of an audit tool. Health Place. 2010;16: 776–783. 10.1016/j.healthplace.2010.04.002 20435506 PMC3820999

[173] Burke NM, Chomitz VR, Rioles NA, Winslow SP, Brukilacchio LB, Baker JC. The path to active living: Physical activity through community design in Somerville, Massachusetts. Am J Prev Med. 2009;37: S386–S394. 10.1016/j.amepre.2009.09.010 19944939

[174] Geraghty AB, Seifert W, Preston T, Holm CV, Duarte TH, Farrar SM. Partnership moves community toward complete streets. Am J Prev Med. 2009;37: S420–S427. 10.1016/j.amepre.2009.09.009 19944943

[175] Gomez-Feliciano L, McCreary LL, Sadowsky R, Peterson S, Hernandez A, McElmurry BJ, et al Active living Logan Square: Joining together to create opportunities for physical activity. Am J Prev Med. 2009;37: S361–S367. 10.1016/j.amepre.2009.09.003 19944936

[176] Huberty JL, Dodge T, Peterson K, Balluff M. Activate Omaha: The journey to an active living environment. Am J Prev Med. 2009;37: S428–S435. 10.1016/j.amepre.2009.09.024 19944944

[177] Lee SM, Tudor-Locke C, Burns EK. Application of a walking suitability assessment to the immediate built environment surrounding elementary schools. Health Promot Pract. 2008;9: 246–252. 10.1177/1524839907301403 18344317

[178] Nelson KM. Designing healthier communities through the input of children. J Public Health Manag Pract. 2008;14: 266–271. 10.1097/01.PHH.0000316485.49888.f6 18408551

[179] Schasberger MG, Hussa CS, Polgar MF, McMonagle JA, Burke SJ, Gegaris AJ. Promoting and developing a trail network across suburban, rural, and urban communities. Am J Prev Med. 2009;37: S336–S344. 10.1016/j.amepre.2009.09.012 19944933

[180] Budgen P, Furber S, Gray E, Zask A. Creating active playgrounds in primary schools. Health Promot J Austr. 2007;18: 77–79. 17501716 10.1071/he07077

[181] Eves FF, Olander EK, Nicoll G, Puig-Ribera A, Griffin C. Increasing stair climbing in a train station: The effects of contextual variables and visibility. J Environ Psychol. 2009;29: 300–303.

[182] Duderstadt KG. State legislators lead fight against childhood obesity. J Pediatr Health Care. 2009;23: 269–271. 10.1016/j.pedhc.2009.04.009 19559995

[183] Boehmer TK, Luke DA, Haire-Joshu DL, Bates HS, Brownson RC. Preventing childhood obesity through state policy. predictors of bill enactment. Am J Prev Med. 2008;34: 333–340. 10.1016/j.amepre.2008.01.003 18374247

[184] Gostin LO. Law as a tool to facilitate healthier lifestyles and prevent obesity. JAMA. 2007;297: 87–90. 17200479 10.1001/jama.297.1.87

[185] Grantmakers in Health. Reversing the obesity epidemic: Policy strategies for health funders Issue Brief. Washington, DC: Grantmakers in Health 2007. 17621687

[186] Huang TTK, Horlick MN. Trends in childhood obesity research: A brief analysis of NIH-Supported efforts. The Journal of Law, Medicine & Ethics. 2007;35: 148–153. 10.1111/j.1748-720X.2007.00119.x 17341223

[187] King AC, Jeffery RW, Fridinger F, Dusenbury L, Provence S, Hedlund SA, et al Environmental and policy approaches to cardiovascular disease prevention through physical activity: Issues and opportunities. Health Educ Q. 1995;22: 499–511. 8550373 10.1177/109019819502200407

[188] Watson M, Dannenberg AL. Investment in safe routes to school projects: Public health benefits for the larger community. Prev Chronic Dis. 2008;5: A90–A90. 18558040 PMC2483559

[189] Cardon GM, Van Acker R, Seghers J, De Martelaer K, Haerens LL, De Bourdeaudhuij IMM. Physical activity promotion in schools: Which strategies do schools (not) implement and which socioecological factors are associated with implementation? Health Educ Res. 2012;27: 470–483. 10.1093/her/cys043 22388742

[190] French SA, Story M, Jeffery RW. Environmental influences on eating and physical activity. Annu Rev Public Health. 2001;22: 309–335. 11274524 10.1146/annurev.publhealth.22.1.309

