# Supplementary figures and images for: Physical Activity Design Guidelines for School Architecture
Source: PLoS One. 2015 Jul 31;10(7):e0132597. doi: 10.1371/journal.pone.0132597 (PMC4521876; doi:10.1371/journal.pone.0132597)

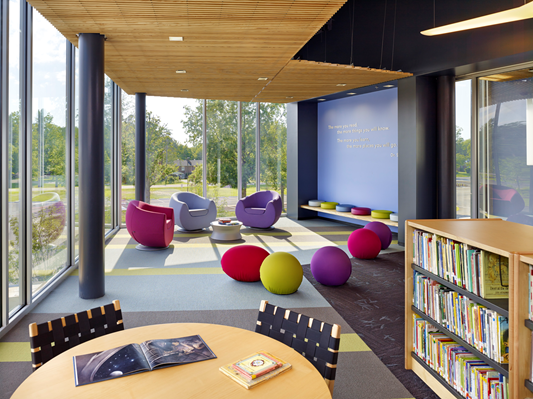

Supplement: S1 Fig — Woodson Education Complex, Dillwyn, Virginia. Much of the school interior includes ample glazing for natural lighting and views of nature. (Photo Credit: Alan Karchmer/VMDO Architects). (TIF) [file pone.0132597.s001.tif]

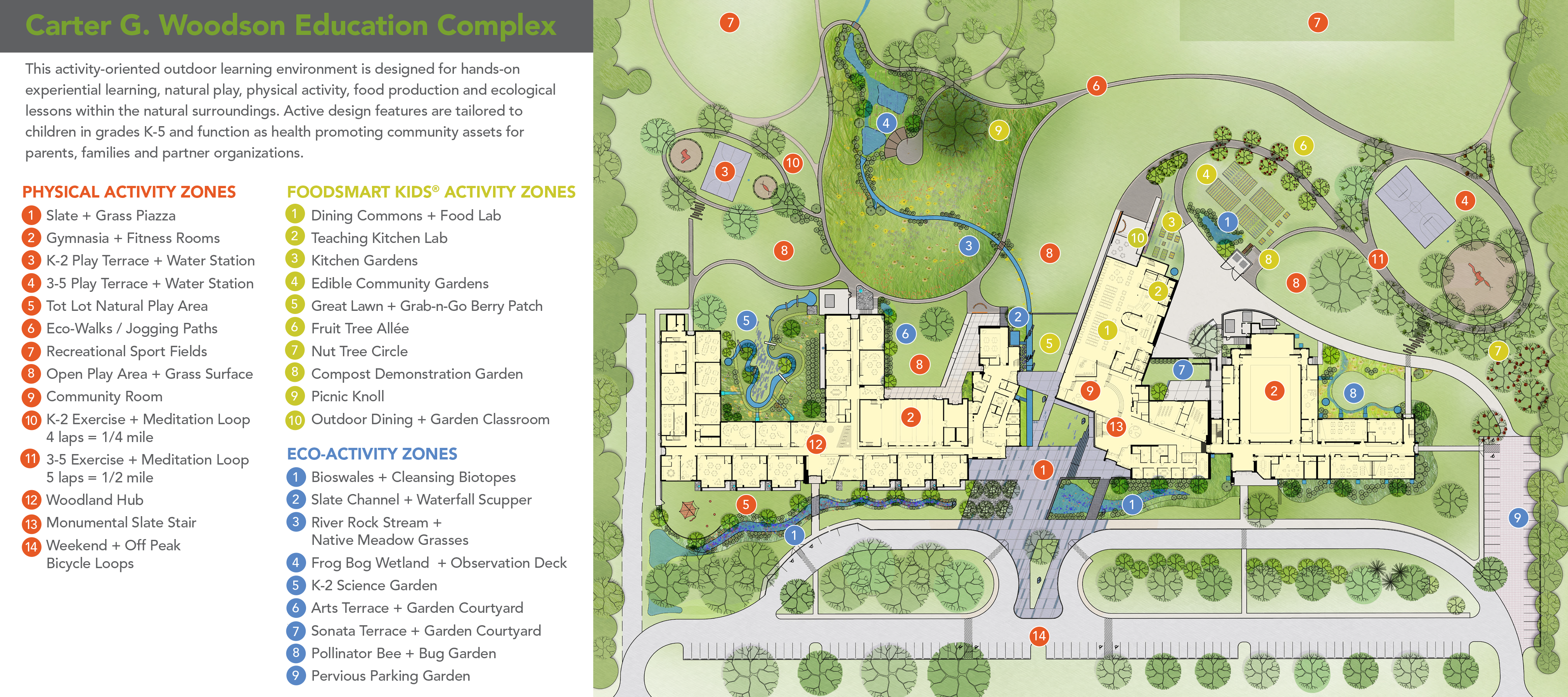

Supplement: S2 Fig — Woodson Education Complex, Buckingham County, Virginia. The design promotes bouts of walking during the school day, and includes many varieties of age-appropriate physical activity opportunities. (Image Credit: VMDO Architects/Water Street Studios). (TIF) [file pone.0132597.s002.tif]

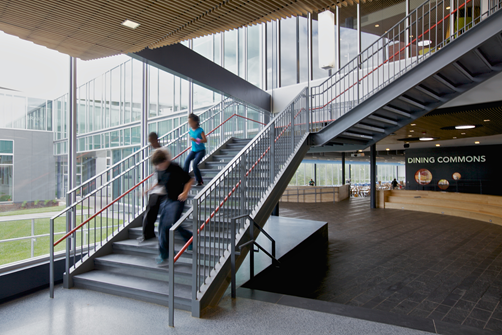

Supplement: S3 Fig — Woodson Education Complex, Dillwyn, Virginia is located near the entry and interior community commons and gathering area. An elevator is available, but located less conspicuously. (Photo Credit: Tom Daly/VMDO Architects). (TIF) [file pone.0132597.s003.tif]

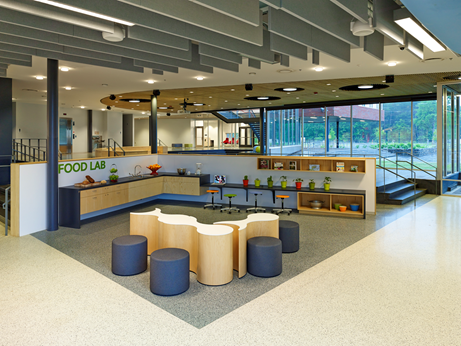

Supplement: S4 Fig — Woodson Education Complex, Dillwyn, Virginia include a food lab, located in close proximity to the community commons with amphitheatre seating, the dining commons, corner bakery, monumental stair, and entry, all with ample light and outdoor views. (Photo Credit: Alan Karchmer/VMDO Architects). (TIF) [file pone.0132597.s004.tif]

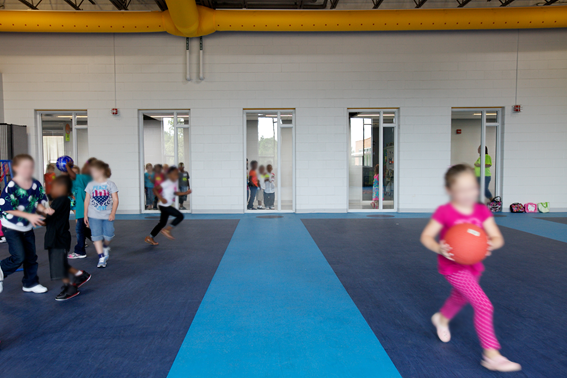

Supplement: S5 Fig — Woodson Education Complex, Dillwyn, Virginia, encourage students to be active. (Photo Credit: Tom Daly/VMDO Architects). (TIF) [file pone.0132597.s005.tif]

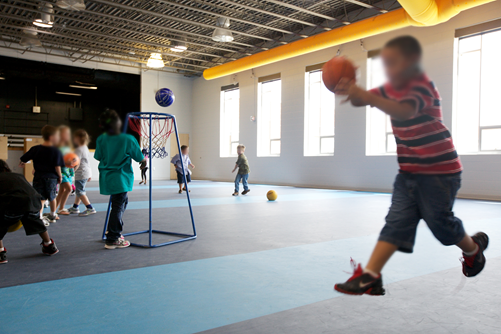

Supplement: S6 Fig — Woodson Education Complex, Dillwyn, Virginia, colored floor markings, including wide bands and circles, delineate spaces for various types of simultaneous activities. (Photo Credit: Tom Daly/VMDO Architects). (TIF) [file pone.0132597.s006.tif]

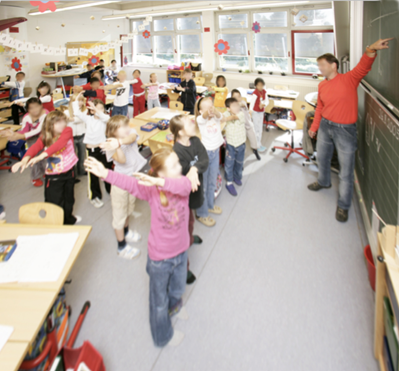

Supplement: S7 Fig — (Photo Credit: Dieter Breithecker/Institute for Posture and Mobilisation Support). (TIF) [file pone.0132597.s007.tif]

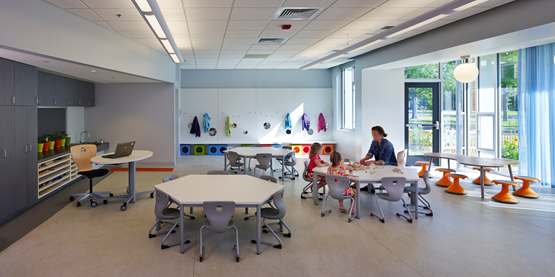

Supplement: S8 Fig — Woodson Education Complex, Dillwyn, Virginia includes dynamic seating and trapezoid-shaped tables that adapt to multiple configurations. The classroom also connects directly to an outdoor play area with rain garden features. (Photo Credit: Alan Karchmer/VMDO Architects). (TIF) [file pone.0132597.s008.tif]

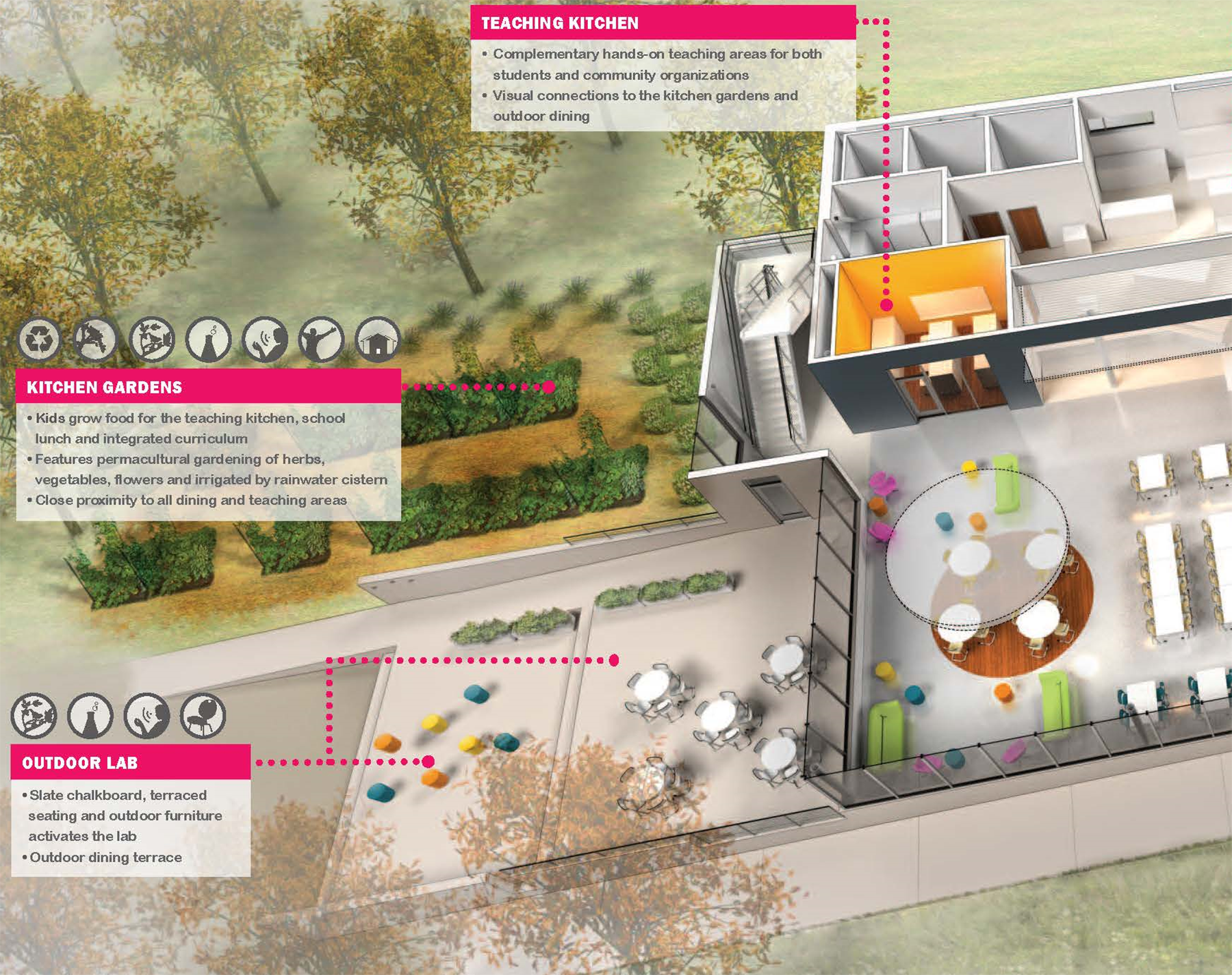

Supplement: S9 Fig — Woodson Education Complex, Dillwyn, Virginia is adjacent to the vegetable and herb garden, edible orchard, interior dining commons, and kitchen lab. A nature trail that runs throughout the school grounds connects to the garden area. (Rendering Credit: VMDO Architects). (TIF) [file pone.0132597.s009.tif]

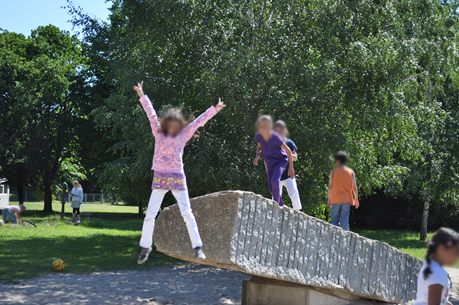

Supplement: S10 Fig — Water is readily available. Here, the students run up an incline and jump off, enjoying the feeling of weightlessness. (Photo Credit: Dieter Breithecker/Institute for Posture and Mobilisation Support). (TIF) [file pone.0132597.s010.tif]

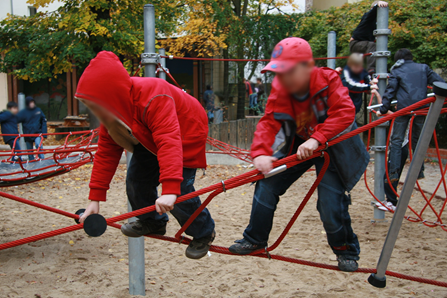

Supplement: S11 Fig — Here, children organize by way of managing hindrances. (Photo Credit: Dieter Breithecker/Institute for Posture and Mobilisation Support). (TIF) [file pone.0132597.s011.tif]

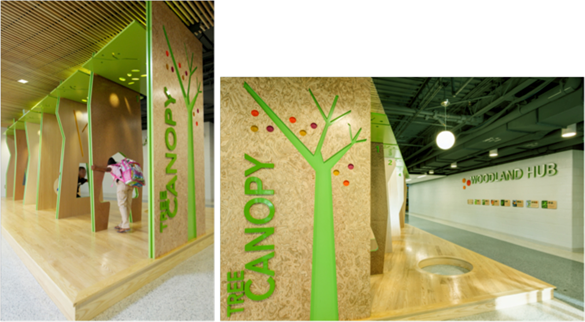

Supplement: S12 Fig — Woodson Education Complex, Dillwyn, Virginia, is intended to entice interactive and active teaching moments and educates about types of trees native to Virginia. (Photo Credits: Tom Daly (left)/Andrea Hubbell (right)/VMDO Architects). (TIF) [file pone.0132597.s012.tif]

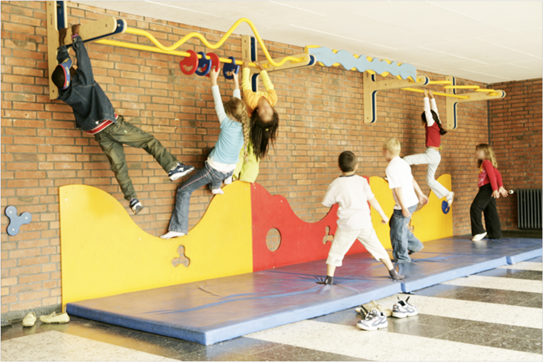

Supplement: S13 Fig — (Photo Credit: Dieter Breithecker/Institute for Posture and Mobilisation Support). (TIF) [file pone.0132597.s013.tif]

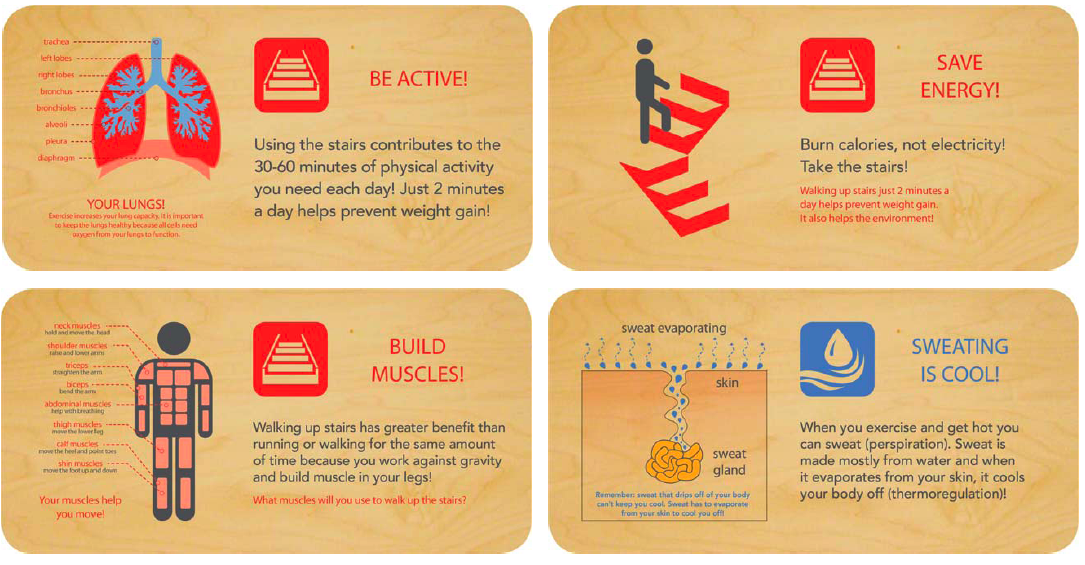

Supplement: S14 Fig — Woodson Educational Complex, Dillwyn, Virginia educates children about the benefits of being physically active. (Image Credit: VMDO Architects). (TIF) [file pone.0132597.s014.tif]

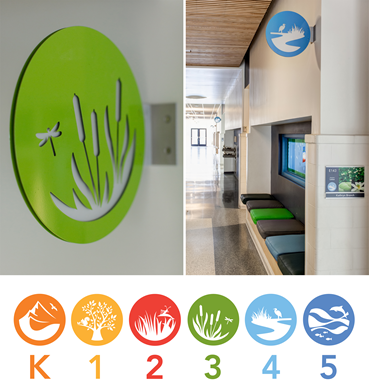

Supplement: S15 Fig — Woodson Educational Complex, Dillwyn, Virginia associates a specific color with each grade level, and engages children to interact visually and physically with educational content. (Image Credit: VMDO Architects). (TIF) [file pone.0132597.s015.tif]

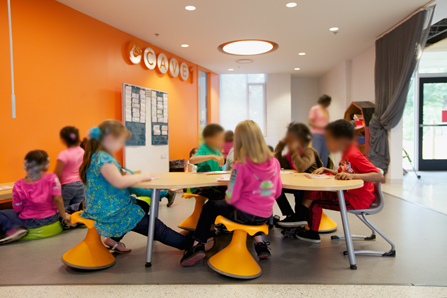

Supplement: S16 Fig — Woodson Educational Complex in Buckingham County, Virginia include dynamic furniture such as these stools with curved bases. (Photo Credit: Tom Daly/VMDO Architects). (TIF) [file pone.0132597.s016.tif]
