# Supplementary material for: Physical Activity Design Guidelines for School Architecture
Source: PLoS One. 2015 Jul 31;10(7):e0132597. doi: 10.1371/journal.pone.0132597 (PMC4521876; doi:10.1371/journal.pone.0132597)
Supplement: S1 Table — (DOCX) [file pone.0132597.s017.docx]

Supplemental Table. Literature Summary.

| **Refe-rence #** | **Author** | **Title** | **Study Design** | **Sample** | **Approach** | **Key Measures** | **Main Findings** | **Strength of Evidence** |
| --- | --- | --- | --- | --- | --- | --- | --- | --- |
|  |  |  |  |  |  |  |  |  |
| 92 | Anthamatten et al. 2011 | An assessment of schoolyard renovation strategies to encourage children’s physical activity | Cross-sectional | Students (n=2,718) age 6-11 at 9 Denver-area schools in underserved neighborhoods (total school enrollment N=3,688) | School-level comparison of utilization and physical activity at Learning Landscapes schoolyards, recently constructed and with older construction, and unrenovated schoolyards.  Learning Landscapes schoolyards included gateways, shade structures, gardens, student and public art | Number of users, percentage of children engaged in MVPA (SOPLAY) | Utilization of Learning Landscapes schoolyards was greater than other schools;  greatest difference between newly constructed and unrenovated schoolyards.  No significant differences in MVPA between schoolyards.  Boys exhibited greater utilization and more vigorous PA in schoolyards overall, compared to girls | Moderate |
| 127 | Babey et al. 2009 | Sociodemographic, family, and environmental factors associated with active commuting to school among US adolescents | Cross-sectional | Youth (n=3,451) age 12-17 across California | Analysis of data from the 2005 California Health Interview Survey to explore associations between socio-demographic, family, and environmental factors and active commuting to school | Active commuting category, numerous socio-demographic, family, and environmental measures | Odds of active commuting to school were higher for those living in urban areas, living closer to school, males, Latinos, from lower-income families, attending public school, without an adult present at home after school, and with parents who knew little about their whereabouts after school | Moderate |
| 142 | Benden et al. 2011 | The impact of stand-biased desks in classrooms on calorie expenditure in children | Cluster RCT | Students (n=58) in 4 1^st^ grade classrooms at 1 ethnically diverse rural Texas school | Random assignment of classrooms to treatment and comparison scenarios for comparison; treatment classrooms received stand-biased desks; 2 5-day intervals of measurement at pre-and post-intervention time points | Body-Bugg armband-measured caloric expenditure | Treatment group experienced significant increases in caloric expenditure during class time vs. the comparison group | Moderate |
| 143 | Benden et al. 2012 | Within-subjects analysis of the effects of a stand-biased classroom intervention on energy expenditure | Longitudinal within-subject, pre/post intervention | Students (n=9) age 6-8 at 1 rural Texas elementary school | 2 consecutive 5-month trials, one in the fall in a classroom with traditional desks, and one in the spring after the entire classroom had been equipped with stand-biased desks; analysis of within-subject differences pre- and post-intervention | Body-Bugg armband-measured caloric expenditure, steps per minute, teacher-reported observed behaviors | Within-subject energy expenditure increased significantly in the intervention scenario with stand-biased desks  Teachers reported an increase in positive in-class behavior and focus on school activities in the intervention scenario | Moderate |
| 144 | Blake et al. 2012 | Using stand/sit workstations in classrooms: Lessons learned from a pilot study in Texas | Cross-sectional qualitative | Parents and teachers (n=unspecified) whose 1^st^ grade students participated in a trial of stand-biased desks in a rural Texas school classroom | Summary of feedback on classroom and behavior observations from parents and teachers, and feedback from students | Observations about desk adjustment, stool use, student conditioning period, and unanticipated effects | Adjustable stand-biased desks, footrests, and stools require more set-up effort than traditional furniture  Although students were told they could use stools or stand at their desks, by the fourth intervention week, more than two-thirds of students had stopped using the stool and removed it from their workstations  Peer influence played a role in conditioning students to the desks, as it became ‘cool’ to stand  Teachers reported an unanticipated positive effect of the intervention on students’ attention and focus | Preliminary |
| 123 | Boarnet et al. 2005 | Evaluation of the California Safe Routes to School legislation: Urban form changes and children’s active transportation to school | Cross-sectional | Parents (n=1,244) of students at 10 California schools within ¼ mile of California Safe Routes to School (SR2S) sites | Analysis of survey data to examine urban form changes, such as installation or widening of bicycle lanes, sidewalks, and crosswalks from SR2S projects, and children’s active transportation to school; comparison of survey responses in 2 groups, parents of children who passed SR2S project on usual route to school, and those whose children did not pass SR2S site; inclusion of retrospective questions to assess change | Retrospective and current parent-reported active commuting to school | Based on parent responses, children who passed SR2S projects on their usual routes to school were more likely to have increased their active travel to school than those who did not pass a SR2S site | Moderate |
| 106 | Boldemann et al. 2006 | Impact of preschool environment upon children’s physical activity and sun exposure | Cross-sectional | Students (n=197) age 4-6 at 11 preschools in Stockholm, Sweden | Data collection via environmental assessment, parent questionnaire, staff questionnaire  (validity and reliability confirmed), and school-time PA measures of children; analysis of associations between environmental variables and children’s PA and UV exposure | Child BMI, environmental factors, pedometer-measured steps, dosimeter-measured UV radiation | Children’s mean step count was higher in environments with trees, shrubbery, and broken ground, vs. delimited environments with little vegetation  UV exposure was lower in environments with trees, shrubbery, and broken ground  No differences between girls and boys | Moderate |
| 157 | Boutelle et al. 2004 | Using signs, artwork, and music to promote stair use in a public building | Cross-sectional | Users of 1 university building | Independent cross-sectional design with pre- and post-intervention data collection  Intervention 1: Signs with health message  Intervention 2: Addition of music and artwork | Percentage of individuals using stairs vs. elevators | Increased stair use with music-artwork intervention  No increase in stair use with sign intervention only | Preliminary |
| 128 | Braza et al. 2004 | Neighborhood design and rates of walking and biking to elementary school in 34 California communities | Cross-sectional | Students (n=2,993) age 9-11 from 105 5^th^ grade classrooms at 34 California public elementary schools | Based on teacher-collected student survey data, U.S. Census data, and California Department of Education data, evaluated the relationships between neighborhood design and rates of student walking and cycling to school | Neighborhood-level measures including density, street network connectivity; School-level measures including school size/enrollment, proportion of students walking or cycling to school | Higher population density and larger school size associated with higher walking and cycling rates, controlling for confounders  Pairwise correlation between number of intersections per street mile and walking/cycling rates did not hold in regression modeling | Moderate |
| 93 | Brink et al. 2010 | Influence of schoolyard renovations on children’s physical activity: The Learning Landscapes program | Cross-sectional | Students (n=2,718) age 6-11 at 9 Denver-area schools in underserved neighborhoods (total school enrollment N=3,688) | Independent cross-sectional comparison of student physical activity at different types of schoolyards, and in different schoolyard surface conditions | Type of schoolyard, schoolyard surface condition, student time in sedentary, moderate, and vigorous PA (SOPLAY), student energy expenditure (SOPLAY calculation) | Utilization of Learning Landscapes schoolyards was greater than comparison schools  Energy expenditure per scan (school level) higher at Learning Landscapes schools vs. comparison schools  Boys’ and girls’ activity rates greater on soft surfaced, structured areas at Learning Landscapes vs. control schools  Boys’ activity rates greater on hard surface unstructured areas at Learning Landscapes vs. control schools | Moderate |
| 75 | Buliung et al. 2009 | Active school transportation in the greater Toronto area | Cross-sectional | Independent population samples (n=2,393-10,670) from the Toronto metropolitan area, at time points between 1986 and 2006 | Analysis of temporal and spatial trends in students active transportation to school | Urban vs. suburban neighborhood, Proportion of active transportation to school at time points,  Children’s age groups | Between 1986 and 2006, walking proportion of school trips declined significantly for both 11-13 year olds and 14-15 year olds  In 2006, 11-13 year olds walked to school less in the suburbs than in urban Toronto  In 2006, 14-15 year olds walked less, but used public transit more, in urban Toronto vs. the suburbs | Moderate |
| 141 | Cardon et al. 2004 | Sitting habits in elementary school children: A traditional vs. a “moving” school | Cross-sectional | Students (n=47) age 8 at 2 schools: a ‘moving school’ in Germany, a traditional school in Belgium | Comparison of physical activity and posture between students in ‘moving’ and traditional school groups  Moving school included dynamic furniture and integration of movement in classroom lessons | Accelerometer-measured PA, postural measures, duration and frequency of sitting | Students at the moving school sat statically less, walked around more, exhibited better posture, had lower prevalence of back pain, and had higher PA levels | Moderate |
| 85 | Cardon et al. 2009 | Promoting physical activity at the pre-school playground: The effects of providing markings and play equipment | Cluster RCT | Students (n=583) age 4-5 at a convenience sample of 40 Belgian public schools | Random assignment of schools to 4 conditions: (1) provision of play equipment, (2) markings painted on playgrounds, (3) provision of play equipment plus markings painted, (4) no change/control; Data collection at pre-and post-intervention time points | Accelerometer-measured activity levels, recess time in MVPA and sedentary activity | No significant impact of playground interventions on either recess sedentary time or time in MVPA | Strong |
| 137 | Cohen et. al 2006 | Proximity to school and physical activity among middle school girls: the Trial of Activity for Adolescent Girls Study | Cross-sectional | Female students (n=1,554) in middle school enrolled in the multi-state TAAG study | Examination of relationship between distance to school and PA among girls, controlling for potential confounders | Shortest distance between home and school along street network, accelerometer-measured MET-weight MVPA | Distance to school was inversely associated with MET-weight MVPA  For each incremental mile from school, girls engaged in an average of 13 fewer MET-weighted minutes per week | Moderate |
| 96 | Cohen et al. 2008 | School design and physical activity among middle school girls | Cross-sectional | Female students (n=1,566) in middle school who were enrolled in the multi-state Trial of Activity for Adolescent Girls (TAAG) | Cross-sectional analysis of school environment factor associations with levels of PA | Size of school building footprint and school grounds, count of active outdoor amenities, in-school accelerometer-measured MET-weight MVPA and light PA | Number of outdoor PA facilities was positively associated with MVPA, but mediated by weather  Outdoor field size was not associated with PA |  |
| 90 | Colabianchi et al. 2009 | Utilization and physical activity levels at renovated and unrenovated school playgrounds | Cross-sectional | Users of 20 school playgrounds (10 renovated and 10 unrenovated) in Cleveland | School-level analysis of usage and PA at renovated vs. unrenovated playgrounds, schools matched on school and neighborhood characteristics, children observed outside of school hours | Usage of playground, proportion of children engaged in MVPA on the playground (SOPLAY) | Higher overall utilization of renovated vs. unrenovated playgrounds  No significant difference between proportion of time spent in MVPA at renovated vs. unrenovated playgrounds | Moderate |
| 91 | Colabianchi et al. 2011 | Features and amenities of school playgrounds: A direct observation study of utilization and physical activity levels outside of school time | Cross-sectional | Users of 20 school playgrounds (10 renovated and 10 unrenovated) in Cleveland | School-level analysis of usage and PA at renovated vs. unrenovated playgrounds, schools matched on school and neighborhood characteristics, children observed outside of school hours, analysis of associations with a playground attributes | Usage of playground, proportion of children engaged in MVPA (SOPLAY), playground attributes from the Environmental Assessment of Public Recreation Spaces assessment tool | At renovated playgrounds, total number of play features positively associated with utilization among adults and girls  Lower cleanliness was associated with lower usage among boys and girls  Coverage and shade for resting features positively associated with utilization among boys  No significant associations between playground attributes and proportion of active children | Moderate |
| 154 | Community Preventive Services Task Force 2010 | Recommendations for use of point of decision prompts to increase stair use in communities | Review | Published studies addressing use of stair point-of-decision prompts | Systematic review of research addressing the impact of point-of-decision prompts for stair use | N/A | Stair point-of-decision prompts may increase stair use  Insufficient evidence to show effectiveness of stairwell enhancements with point-of-decision prompts | Moderate |
| 109 | Cradock et al. 2007 | Characteristics of school campuses and physical activity among youth | Cross-sectional | Students (n=248) in 10 middle schools in the Boston area | Associational analysis of school characteristics from site data collection and secondary data sources in 2004-5, and student physical activity data collected in 1997 for RCT of a school-based intervention | Accelerometer-measured vector magnitude, school characteristics including campus area, play area, and building area per student | Larger school campus area per student, building area per student, and play area per student were positively associated with PA  Mean vector magnitude differences translated to walking 2 additional miles over a week’s time | Moderate |
| 138 | D’Haese et al. 2011 | Criterion distances and environmental correlates of active commuting to school in children | Cross-sectional | Parents (n=696) or 6^th^ grade students in 44 randomly selected classes at Belgian elementary schools | Analysis to determine home to school criterion distances at which at least 85% of active school commuters lived  Subsequent analysis to identify correlates of active commuting within these distances | Neighborhood Environment Walkability Scale for Youth (NEWS-Y) subscales, parent-reported child active commuting to school, distance from home to school | 59.3% of total sample actively commuted to school  Criterion distances set at 1.5 kilmeters for walking and 3.0 kilometers for cycling  At home to school distance of 2.01-2.50 kilometers, number of passive commuters exceeded active commuters  Among active commuters, longer distance to school associated with more cycling vs. walking | Moderate |
| 2 | Dordel and Breithecker 2003 | Bewegte Schule als Chance einer Förderung der Lern- und Leistungsfähigkeit | Cluster matched controlled trial | Students (n= 56) in 3^rd^ grade from 3 classrooms at a German elementary school | Compared students’ concentration at 3 times during the school day based on 3 levels of school-based environment-influenced PA: (A) typical class and school environment; (B) class with space and encouragement to do moving activities and a schoolyard with features to inspire exertion; (C) class that included an active learning pedagogy, a dynamic sitting and flexible furniture environment, and a schoolyard like group B | Concentration performance measured via attention stress-test | Academic performance in the class with moving activities and active schoolyard (B) was better than in the typical class and school environment (A) during the school morning  Academic performance in the classroom with ergonomic furniture, moving activities, and active schoolyard (C) were significantly better than both (A) and (B)  Group (A) in the typical school environment recorded a significant decline in academic performance at later times of day | Moderate |
| 57 | Durant et al. 2009 | Relation of school environment and policy to adolescent physical activity | Cross-sectional | Students (n=165) age 18 in 3 U.S. cities | Analysis of survey data for associations between PA and several school environment variables | Self-reported PA, school PA equipment accessibility, field access, after-school supervised PA, days of PE class per week | Access to school fields after school, and days of PE per week positively correlated with overall PA  PA equipment and after-school supervised PA not associated with overall PA | Moderate |
| 104 | Dyment and Bell 2007 | Active by design: Promoting physical activity through school ground greening | Cross-sectional | Teachers, parents, and administrators (n=105) associated with 59 Canadian schools that had “greened” the school site | Used data from a prior national survey  Analyzed participants’ perspectives as to the impact of school culture and grounds characteristics on students’ PA (content validity confirmed) | Percentages of participants designating design and culture factors of school grounds as encouraging or discouraging PA | Adequate space, diverse play opportunities, and interaction with natural elements deemed important in stimulating active play  Children were perceived to be more active with opportunities for garden or green space care, and when rules and supervision allow open-ended play | Moderate |
| 105 | Dyment and Bell 2008 | Grounds for movement: Green school grounds as sites for promoting physical activity | Cross-sectional | Teachers, parents, and administrators (n=105) associated with 59 Canadian schools that had “greened” the school site | Used data from a prior national survey  Analyzed participants’ perspectives as to the impact of school culture and grounds characteristics on students’ PA (content validity confirmed) | Participant impressions of impact of school ground greening on children’s PA | School ground greening seen as diversifying children’s play repertoire, inviting children to jump, climb, dig, lift, role play, etc., and potentially encouraging children’s PA by increasing non-competitive and open-ended play at school | Moderate |
| 125 | Eyler et al. 2008 | Policies related to active transport to and from school | Cross-sectional qualitative | Adult stakeholders (n=69), including teachers, principals, parents, local community organizers, school and city officials, and public safety representatives, at 9 elementary schools in 7 states | Qualitative analysis of school stakeholder interview data regarding school-related policies and student active transport to school | Explored potential factors and policies related to students | Identified 2 distinct aspects of school policies related to active transport to school: (1) influential factors, and (2) policy actions  Influential factors included sidewalks, crosswalks and crossing guards, personal safety concerns, advocacy group involvement  Policy actions included school speed zones, drop-off and no transport zones, school siting, school start and dismissal time | Moderate |
| 86 | Farley et al. 2007 | Safe play spaces to promote physical activity in inner-city children: Results from a pilot study of an environmental intervention | Cluster matched controlled trial | Children (n=710) using 2 school playgrounds after school hours in New Orleans; students (n=465) in grades 2-5 at participating schools | Direct observation of school playground use and PA in an intervention school with an open playground and attendants, vs. a comparison school site, survey of sedentary time | Direct observation usage counts, and PA levels using (modification of SOPLAY),  sedentary time per school-based survey | Number of children outdoors and physically active was higher in the intervention neighborhood, and there were concomitant declines in reported sedentary indoor activities | Moderate |
| 102 | Fein et al. 2004 | Perceived environment and physical activity in youth | Cross-sectional | Students (n=610) in grades 9-12 at 4 rural Canadian high schools | Based on self-report questionnaire, analysis of associations between perceived availability and importance of physical environment resources, and PA | Perceived physical environment resources availability, Perceived physical environment resources importance, self-reported PA | Perceived higher importance of the school environment PA resources (e.g., gym space allows me to do activities, sport/exercise equipment works well, school athletic facilities are accessible, etc.) was associated with PA | Moderate |
| 110 | Fernandes et al. 2010 | Facility provision in elementary schools: Correlates with physical education, recess, and obesity | Cross-sectional | Students (n=8,935) in 5^th^ grade at schools across the U.S, with oversampling of racial/ethic minorities and attendees of private schools | Analysis of associations between demographic and location variables, and availability and adequacy of gymnasium and playground; analysis of associations between facility and location characteristics, and physical education and recess time; used data from the Early Childhood Longitudinal Survey Kindergarten Cohort | Multiple variables including child weight status, degree of urbanization, climate zone, availability and adequacy of gymnasium, availability of adequate playground, physical education time, recess time | Students from underserved backgrounds more likely to attend a school with poorer gymnasium and playground provision  Gymnasium availability associated with additional 8.3 minutes of PE per week, and additional 25 minutes in humid climate zones  No significant results of playground and gymnasium adequacy in relation to PE and recess time, or in relation to obesity trajectory | Moderate |
| 139 | Fitzhugh et al. 2010 | Urban trails and physical activity: A natural experiment | Longitudinal pre/post intervention with comparison group | Children, adolescents, and adults living in 3 Knoxville, Tennessee neighborhoods | Comparison of changes over 2 years in physical activity in the intervention neighborhood that was retrofitted with an urban trail, and in 2 comparison neighborhoods | Counts of directly observed PA,  Counts of active transport to school | Counts of physical activity increased in the intervention neighborhood retrofitted with an urban trail, and decreased in the comparison neighborhood  No intervention effect on counts of active commuting to school | Moderate |
| 95 | Fjørtoft et al. 2010 | Schoolyard physical activity in 14-year-old adolescents assessed by mobile GPS and heart rate monitoring analysed by GIS | Cross-sectional | Students (n=81) age 14, in 9^th^ grade at 2 Norwegian schools | Spatial tracking of children’s movements and monitoring of heart rates during outdoor activities at school lunch break over a several day period; mapping of average heart rates to spatial grids with conversion to GIS wire graphs; confirmation that BMI of sample was comparable to national data | Students’ chest belt-measured heart rate, recorded via GPS device; students’ GPS-measured movements; proportion of time spent in LPA, MVPA, VPA, per heart rate conversion | At both schools, 70% of students’ break time was allocated to low levels of PA  Highest levels of PA occurred at a handball goal area, with higher intensity in girls vs. boys | Moderate |
| 153 | Ford and Torok 2008 | Motivational signage increases physical activity on a college campus | Cross-sectional with intervention | Users of 1 college campus building | Independent cross-sectional analysis to compare stair use before and after signage intervention | Stair use at baseline, with motivational signage intervention, and after signage removed | Motivational signs significantly increased stair use, which was maintained one week after signs were removed | Preliminary |
| 65 | Garcia et al. 2014 | Comparison of stable and dynamic school furniture on physical activity and learning in children | Longitudinal within-subject, 2 exposures | Students (n=12) in 1^st^-6^th^ grade at a rural Virginia primary and elementary school | Children participated in 2 conditions, stable vs. dynamic furniture, presented in balanced order; within-subject analysis of differences in PA, energy expenditure, and learning between the two conditions | Accelerometer-measured activity counts, indirect calorimetry device-measured energy expenditure, answers to questions on a brief lecture and age-appropriate math problems | Average activity counts greater in the dynamic vs. stable furniture condition  No significant differences in energy expenditure or percentage of questions and problems answered correctly  75% of participants reported a preference for sitting in the dynamic vs. stable furniture | Preliminary |
| 132 | Giles-Corti et al. 2011 | School site and the potential to walk to school: The impact of street connectivity and traffic exposure in school neighborhoods | Cross-sectional | Students (n=1,480) in school years 5-7 and their parents (n=1,332) at 25 Australian primary schools | Analysis of associations of children walking to school with neighborhood walkability, based on street connectivity and traffic exposure, within 2 km of schools | School-specific walkability index, pedshed (ratio of pedestrian network area to total area), vehicular traffic exposure, measured weight status, frequency of walking to school | Regular walking to school was greater in high walkable neighborhoods with high street connectivity and low traffic volumes  Regular walking to school was less likely in neighborhoods with high connectivity and high traffic | Moderate |
| 130 | Harrison et al. 2011 | Environmental correlates of adiposity in 9-10 year old children: Considering home and school neighbourhoods and routes to school | Cross-sectional | Children (n=1,995) age 9-10 in the UK | Analysis of data from the SPEEDY (Sport, Physical activity and Eating behavior: Environmental Determinants in Young people) to investigate environmental correlates of weight status in the home neighborhood, school neighborhood, and modeled route between home and school | Fat mass index (FMI), characteristics of areas around homes, schools, and routes to school | Among girls, higher proportion of accessible open land and lower mix of land uses around school associated with higher FMI  Among active traveler boys, major roads in school area associated with lower FMI  Among non-active traveler boys, presence of major roads in home neighborhood associated with higher FMI  No associations between FMI and route characteristics | Moderate |
| 94 | Haug et al. 2010 | The characteristics of the outdoor school environment associated with physical activity | Cross-sectional | Students (n=16,471) in primary grades 4-7, and secondary grades 8-10 at Norwegian schools | Analysis of associations in data collected via self-administered questionnaires | School physical environment characteristics, daily physical activity during school breaks | At secondary level:  Boys and girls had higher odds of being physically active at schools with larger number of outdoor facilities, and at schools with a sledding hill vs. those without  Boys had higher odds of being physically active at schools with hopscotch/skipping rope areas, at schools with soccer fields, at schools with playground equipment  No significant results at primary level | Moderate |
| 122 | Heinrich et al. 2011 | Hawai’i’s opportunity for active living advancement (HO’ĀLA): Addressing childhood obesity through Safe Routes to School | Cross-sectional | Parents (n=1,648) of children in 1^st^ and 4^th^ grades from 13 schools in under-resourced communities in Hawai’i | Report of baseline measures for a planned longitudinal study of Safe Routes to School (SR2S) and active commuting and PA; descriptive analysis of parent survey, data from PATH Hawai’i SR2S Toolkit and Pedestrian Environment Data Scan | Parent-reported travel modes to and from school, Distance from home to school, Traffic counts and safety on routes,  Physical condition of street segments on routes | Among the 5 schools in neighborhoods and 8 in rural settings, few children walked or biked to school, and most were driven to and from school by parents | Preliminary |
| 111 | Hobin et al. 2010 | A multilevel examination of factors of the school environment and time spent in moderate to vigorous physical activity among a sample of secondary school students in grades 9-12 in Ontario, CA | Cross-sectional | Students (n=22,117) in grades 9-12 at 72 Ontario secondary schools | Analysis of associations between student and environment characteristics and student PA, based on student survey and GIS data | Environment- and student-level characteristics, student self-reported time spent in MVPA | School level differences accounted for 3% of the variability in student MVPA; Students of schools with daily PE or provision of alternate room for physical activity spent more time in MVPA than students at schools lacking these resources; As school neighborhood walkability and land-use mix increased, student time spent in MVPA decreased | Moderate |
| 79 | Huberty et al. 2011 | Environmental modifications to increase physical activity during recess | Cross-sectional | Students (n=237) in 3^rd^-6^th^ grade at 4 schools in a Midwestern metropolitan area | One school assigned to each of the following scenarios: (1) Provision of recreational equipment and staff training, (2) Provision of recreational equipment, (3) Provision of staff training, (4) Control/no training or equipment provided  Analysis of associations between scenarios and MVPA outcomes | Accelerometer-measured PA, weight status | Compared with the control, healthy weight boys with equipment and staff training had more MVPA (greatest difference), overweight and obese boys with staff training had more MVPA, overweight and obese girls with equipment and staff training had more MVPA, and healthy weight girls with equipment exhibited less MVPA | Moderate |
| 136 | Kerr et al. 2006 | Active commuting to school: Associations with environment and parental concerns | Cross-sectional | Parents (n=259) of children age 5-18, randomly selected from neighborhoods chosen for variability in neighborhood characteristics and income in Seattle, WA | Analysis of questionnaire data to explore relationships of objective and perceived neighborhood environment characteristics, parent concerns about children’s active commuting to school, with the outcome of active commuting to school | Perceived neighborhood characteristics, GIS- and Census-measured neighborhood characteristics, Parent-reported frequency of child’s active commuting, parental concern scale | Parental concern inversely associated with students’ active commuting  Among high-income neighborhoods, more active commuting in higher vs. lower walkability neighborhoods  Among low-income neighborhoods, no difference in active commuting based on neighborhood walkability  Neighborhood aesthetics independently associated with active commuting | Moderate |
| 140 | Lanningham-Foster et al. 2008 | Changing the school environment to increase physical activity in children | Longitudinal within-subject, 3 exposures | Students (n=40) in 4^th^-5^th^ grades at a Rochester, MN elementary school | Comparison of students’ PA in 3 school environments: traditional school with chairs and desks, activity-permissive open environment called “The Neighborhood,” traditional school with desks that encouraged standing;  cross-sectional comparison with age-matched group on summer vacation | Accelerometer-measured physical activity | PA levels of children while attending school at ‘The Neighborhood” were higher than in both the traditional and stand-biased classroom, and were equivalent to activity levels of the group on summer vacation | Moderate |
| 151 | Lee et al. 2012 | Promoting routine stair use: Evaluating the impact of a stair prompt across buildings | Cross-sectional with intervention | Users of 3 New York City buildings: a 3-story health clinic, an 8-story academic building, and a 10-story housing structure | Independent cross-sectional analysis to compare stair use before and after posting of prompt stating, “Burn Calories, Not Electricity”; measure pre- and immediately post-intervention, with 9 month follow-up at 2 sites | Ascending and descending stair and elevator trips | Increased stair use at all sites after posting of prompt  Relative increases in stair use maintained at the 2 sites with 9 month follow-up | Moderate |
| 152 | Lewis and Eves 2012 | Prompt before the choice is made: Effects of a stair-climbing intervention in university buildings | Cross-sectional with intervention | Users of 4 university buildings | Independent cross-sectional analysis to compare impact of interventions: (1) Motivational signage in elevator, (2) Point-of-choice prompt | Counts of stair users | No effect of motivational signage  Stair climbing increase with the point-of-choice prompt | Moderate |
| 129 | Loucaides 2009 | School location and gender differences in person, social, and environmental correlates of physical activity in Cypriot middle school children | Cross-sectional | Students (n=676) at middle schools in Cyprus | Exploration of possible associations of personal, social, and environmental factors with PA, with intent to understand why obesity and overweight status more prevalent in rural areas | Urban vs. rural school location, numerous personal, social, and environmental factors | Significant interaction effects of female gender and rural location on weekly frequency parent transports child, and lower weekly frequency of sports club attendance  Boys reported play outside more hours per day than girls | Moderate |
| 148 | Ludwig and Breithecker 2008 | Untersuchung zur Änderung der Oberkörperdurchblutung während des Sitzens auf Stühlen mit beweglicher Sitzfläche | Matched controlled trial | Male students (n=10) age 14, in 8^th^ grade at a German school | Comparison of students’ thermal body temperatures, one group using traditional rigid seating and one group using dynamic seating | Trunk body temperature measured by infrared imagery and software thermography | Higher body temperature over 3 school hours in dynamic vs. static seating | Moderate |
| 108 | Martin et al. 2012 | School and individual-level characteristics are associated with children’s moderate to vigorous intensity physical activity during school recess | Cross-sectional | Students (n=408) in 6^th^ grade at 27 Austrialian primary schools | Analysis of associations between children’s recess MVPA and child, school, policy, and socio-cultural factors | Accelerometer-measured PA, multiple individual and environmental factors | Higher daily recess MVPA was associated with newer schools, schools with a higher number of grassed surfaces per child and fewer shaded grass surfaces, and schools with a PE coordinator meeting Australian guidelines | Moderate |
| 97 | Millstein et al. 2011 | Home, school, and neighborhood environment factors and youth physical activity | Cross-sectional | Youth (n=137) age 12-18, and parents (n=104) of children aged 5-11, from San Diego, Boston, and Cincinnati areas | Analysis of associations between environment factors and youth PA, based upon survey data (test-retest reliability confirmed) | Self- or parent-reported PA, Home, Proxy-reported travel information, School and neighborhood environment factors | Count of school PA equipment positively associated with adolescent PA, but not PA of younger children  Some home and neighborhood characteristics associated with PA for children and/or adolescents | Moderate |
| 126 | Mitra et al. 2010 | Spatial clustering and the temporal mobility of walking school trips in the greater Toronto area, Canada | Cross-sectional | Households with 11-13 years olds in the Greater Toronto Area (817,000 trip records) | Analysis of travel data from the Transportation Tomorrow Survey, and urban area classification, based upon spatial and temporal (AM vs. PM) clustering | Spatial and temporal clustering of trips, Urban area classification | Higher spatial clustering of walking in the urban and inner-suburban areas, and in low household income areas  Temporal clustering of walking less likely in inner-suburban and outer-suburban than in urban areas | Moderate |
| 107 | Nicaise et al. 2012 | Evaluation of a redesigned outdoor space on preschool children’s physical activity during recess | Cross-sectional with intervention | Students (n=107) age 4-5 at a university preschool | Collection of data from 2 independent samples at baseline, and several months after an outdoor space redesign intervention;  renovation based on urban naturalism concepts, with plantings and land contours intended to promote discovery and social interaction, and including a looping path, addition of a grassy hill, and removal of 2 play structures to create more open space | Accelerometer-measured PA, ObservationPA (OSRAC-P) | Based on observational data, fewer intervals spent sedentary and more intervals in light PA in the intervention scenario vs. the baseline scenario  Higher odds of observed MVPA with the new looping cycle path, increased playground open space, and the new grass hill  No significant results based on accelerometry data | Moderate |
| 149 | Nicoll et al. 2007 | Spatial measures associated with stair use | Cross-sectional | Users of 10 buildings on 2 university campuses | Analysis of associations between stair use and spatial variables | Stair use measured with infrared monitors, spatial measures, appeal, convenience, comfort, legibility, and safety of stairs | Stair use was associated with shorter travel distance to entrance, higher area and accessibility of stair, area of visual field from stair, fewer turns required from stair to entrance, and most integrated path to stair  No significant association of stair use with appeal, comfort, or safety | Moderate |
| 89 | Nielsen et al. 2010 | Permanent play facilities in school playgrounds as a determinant of children’s activity | Cross-sectional | Students (n=417) age 5-12 at 7 schools in semirural New Zealand communities | Analysis of association between school permanent play facilities and student PA; permanent play facilities defined as physical structures on the school grounds, excluding buildings, used by children for play and/or sports activities, e.g., swings, slides, clusters of trees, playground markings, goals and hoops for ball activities, etc. | Number of permanent play facilities at schools, accelerometer-measured activity counts and MVPA in and outside of school | Number of permanent play facilities in schools ranged from 14 to 35, and was positively associated with PA  With additional permanent play facilities, average accelerometer counts increased both in school and overall  Each additional play facility associated with more time in MVPA both in school and overall | Moderate |
| 155 | Nocon et al. 2010 | Increasing physical activity with point-of-choice prompt: A systematic review | Review | Studies (n=25) | Systematic literature review | N/A | Point-of-choice stair prompts increased rate of stair climbing in escalator settings, but not definitively in elevator settings | Moderate |
| 88 | Ozer 2007 | The effects of school gardens on students and schools: Conceptualization and considerations for maximizing healthy development | Review | Studies (n=5) | Literature review and conceptual framework | N/A | Four studies addressed nutrition or PA outcomes, deemed promising but overall inconclusive  Proposed a conceptual framework for potential impacts of school gardens | Preliminary |
| 133 | Panter et al. 2010 | Attitudes, social support and environmental perceptions as predictors of active commuting behavior in school children | Cross-sectional | Parents/guardians and children (n=2,012) age 9-10 in urban areas, towns, and villages in Norfolk, England | Based on data from the SPEEDY (Sport, Physical activity and Eating behavior: Environmental Determinants in Young people), analysis of associations between active commuting behavior and potential correlates | Active commuting behavior, Child BMI, Attidudinal and social support factors, Neighborhood and route environment characteristics | 40% of children usually walked to school, and 9% cycled  Positive associations between active commuting to school and parental attitudes, lower safety concerns, social support from parents and friends, parent-reported neighborhood walkability  Negative association of distance to school and active commuting moderated by parental attitudes for short distances, and safety for long distances | Moderate |
| 121 | Panter et al. 2010 | Neighborhood, route, and school environments and children’s active commuting | Cross-sectional | Students (n=2,012) age 9-10 at 92 schools in Norfolk county, UK | Associational analysis of active commuting to school with characteristics of neighborhood and route to school, and school environments (assessed via school audit and teacher questionnaires) | Frequency of active commuting to school, GIS measures of neighborhood characteristics and routes to school, School environment factors | Students had lower odds of walking to school with higher directness of route based on route length/direct distance ratio, and lower odds of walking with greater distance  Students had higher odds of walking to school with higher road density, and without a main road on the route | Moderate |
| 160 | Poole | The place for ubiquitous computing in schools: Lessons learned from a school-based intervention for youth physical activity | Longitudinal pre/post intervention | Students (n=1,465) age 11-13 at 37 Title I U.S. middle schools; Survey sample subset: Students (n=577), parents (n=380), teachers (n=19) | Evaluation of PA impact of the American Horsepower Challenge (AHPC), a pedometer-based health game in a designed virtual reality environment | Pedometer-measured steps/day, Game website usage, Survey-reported PA attitudes, social support | Participants’ PA levels increased during the game time period | Moderate |
| 83 | Ridgers et al. 2007 | Long-term effects of playground markings and physical structures on children’s recess physical activity levels | Cluster matched controlled trial | Students (n=470) at 26 elementary schools in deprived areas of a large city in Northwest England | Comparison of PA trends at 15 intervention school playgrounds redesigned with color-coded zones: red for sports, blue for multiple activities, and yellow for quiet play, and physical sports structures and seating were added, vs.  11 comparison schools with no playground intervention | Recess time spent in heart rate telemeter- and accelerometer-measured PA at baseline, 6-week follow-up, and 6-month follow-up | In both the short and longer term, significant positive intervention effects on recess time spent in MVPA and vigorous PA | Strong |
| 103 | Ridgers et al. 2012 | Physical activity during school recess: A systematic review | Review | Studies (n=53) | Systematic review of 1990-2011 literature pertaining to correlates of students’ school recess PA | N/A | 44 variables identified across the socio-ecological framework  Positive associations of recess PA with overall provision of PA facilities, unfixed equipment, and perceived encouragement of PA | Moderate |
| 150 | Ruff et al. 2014 | Associations between building design, point-of-decision stair prompts, and stair use in urban worksites | Cross-sectional | Adult (n=1,348) employees of the City of New York | Analysis of associations between stair use and building environment and individual variables | Self-reported stair use, Building assessment data | Stair prompts, naturally lit stairwells and stairwell visibility associated with increased likelihood of stair use  Higher floor location, total floors in building, female gender, and higher BMI negatively associated with stair use | Preliminary |
| 78 | Sallis et al. 2001 | The association of school environments with youth physical activity | Cross-sectional | Physical activity areas (n=137) at 24 San Diego public middle schools with mean enrollment of 1,081 students | Area-level analysis of observed students’ non-PE PA in defined school areas; modeling of PA associations with and variance explained by environmental variables | Number of participants, students in MVPA (SOPLAY), school environment variables including area type, area size, improvements (e.g., basketball hoops/courts, other sports courts, etc.) | Environmental variables explained 42% of variance in girls’ PA, and 59% of variance in boys’ PA  Improvements and supervision were associated with PA among girls and boys  Supervision was more important indoors vs. outdoors  Among girls, equipment was associated with higher PA outdoors, but not indoors | Moderate |
| 117 | Salmon et al. 2007 | Associations among individual, social, and environmental barriers and children’s walking or cycling to school | Cross-sectional | Parents (n=720) children age 4-13 from capital cities in Australia | Recruitment of parents via random-digit dialing; analysis of associations between potential influential variables and the outcome of children walking or cycling to school | Parent-reported child frequency of walking or cycling to school, Individual, social, and environmental variables | 41% of children walked or cycled to school 1 or more times per week  Significant environmental barriers were “too far to walk” and “no direct route”  Individual barriers such as “no time in the mornings”, and social barriers such as “no other children to walk with” also significant | Moderate |
| 87 | Scott et al. 2007 | Comparing perceived and objectively measured access to recreational facilities as predictors of physical activity in adolescent girls | Cross-sectional | Female students (n=1,367) in middle school enrolled in the multi-state TAAG study |  | Accelerometer-measured MW-MVPA, Number of objectively measured neighborhood PA facilities, Number of perceived neighborhood PA facilities,  Perceived accessibility of PA facilities | Number of neighborhood PA facilities strongly associated with MVPA  Perceptions of number of facilities associate with PA  For each additional PA facility perceived, there was 3% more MW-MVPA | Moderate |
| 114 | Scott et al. 2007 | Weekend schoolyard accessibility, physical activity, and obesity: The Trial of Activity in Adolescent Girls (TAAG) study | Cross-sectional | Female students (n=1,556) in middle school enrolled in the multi-state TAAG study | Analysis of associations between accelerometer-measured PA over one weekend and the number of PA amenities and accessibility in half-mile radii of girls’ residences | Accelerometer-measured Met Weight-MVPA, PA facilities and accessibility within defined residential areas, BMI | Number of inaccessible school-based facilities was associated with higher BMI  No association of school facility availability and MW-MVPA | Moderate |
| 120 | Silva et al. 2011 | Active commuting: Prevalence, barriers, and associated variables | Cross-sectional | Students (n=1,672) age 11-17 in Brazil | Analysis of self-reported data from a questionnaire about active commuting to school, PA data from a diary method, and sedentary behaviors, and measured fitness and body composition data | Active or passive per self-reported active commuting to school, Low vs. medium/high energy expenditure based diary PA, Hours/day of TV and computer use, BMI, Cardiovascular fitness, Environmental variables | 62.5% of students actively commuted to school  Lower prevalence ratio of active commuting among students of private schools and students living further from schools  Lower prevalence ratio of active commuting with greater time spent commuting  Barriers to active commuting were distance, crime/danger, and traffic  No associations identified with body composition variables | Moderate |
| 98 | Skala et al. 2012 | Environmental characteristics and student physical activity in PE class: Findings from two large urban areas of Texas | Cross-sectional | Students (n=6,740) in 211 3^rd^, 4^th^ and 5^th^ grade PE classes in 74 Texas public schools | Analysis of associations between environmental characteristics and class-level PA | MVPA (SOFIT), Environmental variables including class size, class time, class location, lesson contexts | All environmental variables positively associated with MVPA, except for teacher gender  Children’s MVPA negatively associated with class time and class size, and positively associated with outdoor class location and active lesson context | Moderate |
| 82 | Stratton and Mullan 2005 | The effect of multicolor playground markings on children’s physical activity level during recess | Cluster matched controlled trial | Students (n=240) at 8 schools: 2 early primary (student age 4-7) and 2 late primary ( student age 7-11) schools in Northeast Wales, and 2 early primary and 2 late primary control schools in Northwest England | Analysis of the impact of multicolor playground markings on student PA based on pre- and post-intervention measures; Welsh schools received playground intervention, and English schools served as controls;  schools matched by playground dimensions and student socio-economic status; random selection of participants within school populations | Recess time spent in heart rate telemeter-measured MVPA and vigorous PA | Painting of playground markings in the intervention schools increased time spent in MVPA and vigorous PA, at least in the short term | Strong |
| 124 | Timperio et al. 2006 | Personal, family, social, and environmental correlates of active commuting to school | Cross-sectional | Parents of students (n=235) age 5-6 and students (n=677) age 10-12 from 19 elementary schools in Melbourne, Australia | Self-administered questionnaires to parents of younger children, and self-administered questionnaires to 10-12 year olds; analysis to identify correlates of active commuting (walking or cycling) to school | Reported frequency of student active commuting to school, weight status, multiple neighborhood and school environment, family, social, and individual potential correlates | In both age groups, negative correlates of active commuting included parental perception of few children in neighborhood, no lights or crossings on route to school, and a busy road barrier on the route to school  Among both age groups, children more likely to commute actively if route to school <800 meters  Among younger children, a steep incline on the route to school negatively associated with active commuting  Among older children, good connectivity on route to school negatively associated with active commuting  No associations between perceived energy levels, enjoyment of PA, family factors, or weight status | Moderate |
| 115 | Trilk et al. 2011 | Do physical activity facilities near schools affect physical activity in high school girls? | Cross-sectional | Female students (n=1,394) in 12^th^ grade from 22 South Carolina high schools | Investigation of associations between number of PA facilities within walking distance (.75 mile buffer zone) of school, and self-reported PA behavior | PA from 3-Day Physical Activity Recall (3DPAR), GIS-measured distances between school and PA facilities,  BMI | Overall, girls who attended schools with ≥5 PA facilities within the school buffer zone reported more daily PA than girls with <5 facilities nearby  This finding held for rural schools, but not for girls in urban/suburban schools | Moderate |
| 134 | Van Dyck et al. 2009 | Lower neighbourhood walkability and longer distance to school are related to physical activity in Belgian adolescents | Cross-sectional | Adolescents (n=60) age 12-18 from 120 randomly-selected addresses in a suburban area with low walkability and from an urban area with high walkability, in Belgium | Comparison of PA and active commuting to school between the more and less walkable neighborhoods | Neighborhood Environment Walkability Scale (NEWS) subscores, pedometer- and activity log-measured PA, distance to school | Suburban students, whose schools were further from home, cycled to school more than urban students  No difference in walking to school between suburban and urban students  Marginal significance of higher step count per day among suburban vs. urban students | Moderate |
| 101 | Van Sluijs et al. 2011 | School-level correlates of physical activity intensity in 10-year-old children | Cross-sectional | Students (n=1,908) age 10 at 92 schools in Norfolk, UK | Analysis of associations between school factors and PA intensity based upon a population sample | Accelerometer-measured school-based time in sedentary, moderate, and vigorous PA, 40 school physical and social environment factors | School’s number of sports facilities of at least medium quality associated with greater minutes of VPA | Moderate |
| 84 | Verstraete et al. 2006 | Increasing children’s physical activity levels during recess periods in elementary schools: The effects of providing game equipment | Cluster RCT | Students (n=235) at 7 Belgian elementary schools | Random school assignment to intervention and control groups; analysis of the impact of an intervention providing game equipment on students’ PA during recess and lunch break | Pre- and post-intervention accelerometer-measured MPA and MVPA | Children’s lunch break MVPA and recess MPA increased in the intervention group with game equipment, and decreased in the control group | Strong |
| 118 | Voorhees et al. 2010 | Neighborhood design and perceptions: Relationship with active commuting | Cross-sectional | Female students (n=890) from the multi-state TAAG study, who lived within 1.5 miles of school | Analysis of self-administered survey data about walking behavior and neighborhood, and objective GIS neighborhood data | Self-reported walking to and from school, Perceived characteristics of neighborhood, Objective characteristics of neighborhood | 56% of girls walked to or from school at least 1 day/week  Girls were twice as likely to walk to or from school if they perceived their neighborhoods as safe, and perceived that they had places they liked to walk  Girls were more likely to walk if they lived closer to school, had more active destinations in the neighborhood, and had smaller-sized blocks  White girls walked more frequently than Hispanic or African American girls | Moderate |
| 112 | Wechsler et al. 2000 | Using the school environment to promote physical activity and healthy eating | Review | Studies (n=15 related to school facilities and PA) | Review of literature on aspects of the school environment and their relations to PA and nutrition behaviors, and environmental change interventions promoting PA | N/A | Access to convenient play spaces and facilities positively correlated with young people’s physical activity  Access to a variety of PA facilities may be important | Moderate |
| 64 | Wells et al. 2014 | School gardens and physical activity: A randomized controlled trial of low-income elementary schools | Cluster RCT | Students at 12 New York state elementary schools (survey n=227 across all schools, accelerometry n=124 at 8 schools, direct observation n=117 at 4 schools) | Random assignment of schools to school garden intervention, with 6 control schools waitlisted waitlisted for a garden to be installed after study completion; comparison of school-time PA trends based on measures at baseline, and at 1, 2, and 3 semesters post-intervention, in the 2 scenarios | PA measured by accelerometer, self-report (Girls Health Enrichment Multi-site Study Activity Questionnaire), and direct observation | Self-reported sedentary activity decreased more from baseline to follow-up in the garden schools than in control schools  During the school day, accelerometer-measured MVPA increased more from baseline to follow-up in the garden schools than in control schools  Based on group-level direct observation, children moved more and sat less in outdoor garden-based lesson vs. indoor classroom-based lesson | Strong |
| 77 | Willenberg et al. 2010 | Increasing school playground physical activity: A mixed methods study combining environmental measures and children’s perspectives | Cross-sectional, mixed methods | Students (n=3,006) at 23 primary schools in low socio-economic areas of Melbourne, Australia | Quantitative analysis of associations between student PA and playground characteristics; qualitative analysis of focus groups at a subset of 12 schools including a concept map, group discussion, drawing, and photographic ordering | MPA and VPA (SOPLAY), Playground characteristic, e.g., hard/soft surface, fields with markings/goals, fixed or loose play equipment, no permanent equipment or markings, teacher supervision in setting | Larger proportions of students in VPA with loose equipment, and with teacher supervision, vs. when those were unavailable  Positive associations of fixed play equipment, and hard surfaces with court/play-line markings, with proportion of students in MPA  Qualitatively, children identified fixed play equipment and hard-surfaced courts with play-line markings as invitations to active play | Moderate |
| 135 | Zhu and Lee 2008 | Walkability and safety around elementary schools: Economic and ethnic disparities | Cross-sectional | Neighborhoods in areas around 73 public elementary schools in Austin, TX | Neighborhood-level analysis of disparities in environmental support for walking near elementary schools, based upon secondary data | Neighborhood-level measures including ethnicity proportions, poverty, walkability, crime, visual quality, maintenance, safety, distances to school | Neighborhoods with higher Hispanic student percentage had greater dangers from traffic and crime, and also higher walkability based upon presence of sidewalks, greater density, and mixed land uses  Poor neighborhoods had many adverse street-level conditions, but also shorter distances to school and lower traffic volumes | Moderate |
| 119 | Zhu and Lee 2009 | Correlates of walking to school and implications for public policies: Survey results from parents of elementary school children in Austin, Texas | Cross-sectional | Parents/guardians (n=2,695) of students from 19 elementary schools in Austin, TX | Analysis of survey data to identify correlates of student walking to school | Parent-reported student walking to school, Personal attitudes and behaviors,  School and peer influence factors, Physical environment factors | Among physical environment factors, negative correlates included distance and safety concerns, presence of highways/freeways, convenience stores, office buildings, and bus stops on the route to school  Among personal and social factors, negative correlates of walking to school included parents’ education, car ownership, and school bus availability  Positive correlates included parents’ and children’s positive attitude and regular walking behavior, and supportive peer influences | Moderate |
| 156 | Zimring et al. 2005 | Influences of building design and site design on physical activity: Research and intervention opportunities | Review | Studies (n=unspecified) | Review of studies focused on PA and building and site characteristics; development of ‘working model’ to consider correlates of PA at building and site scales | N/A | Potential for PA impact of building elements such as point-of-choice prompts, site selection, building programming and design  Recommendation for further research, especially in public buildings | Preliminary |
